# Engaging a Non-catalytic Cysteine Residue Drives Potent and Selective Inhibition of Caspase-6

**DOI:** 10.1021/jacs.2c12240

**Published:** 2023-04-27

**Authors:** Kurt S. Van Horn, Dongju Wang, Daniel Medina-Cleghorn, Peter S. Lee, Clifford Bryant, Chad Altobelli, Priyadarshini Jaishankar, Kevin K. Leung, Raymond A. Ng, Andrew J. Ambrose, Yinyan Tang, Michelle R. Arkin, Adam R. Renslo

**Affiliations:** † Department of Pharmaceutical Chemistry, University of California, San Francisco, 600 16th Street, San Francisco, California 94143, United States; ‡ School of Pharmaceutical Sciences, Tsinghua University, Beijing 100084, China; § Chempartner Corporation, 280 Utah Avenue, South San Francisco, California 94080, United States

## Abstract

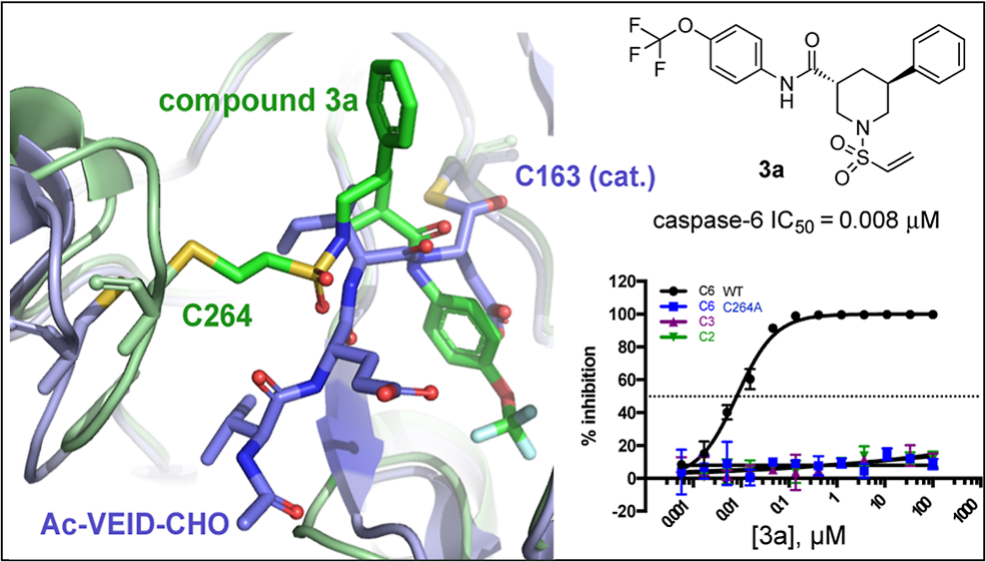

Caspases are a family of cysteine-dependent proteases with important cellular functions in inflammation and apoptosis, while also implicated in human diseases. Classical chemical tools to study caspase functions lack selectivity for specific caspase family members due to highly conserved active sites and catalytic machinery. To overcome this limitation, we targeted a non-catalytic cysteine residue (C264) unique to caspase-6 (C6), an enigmatic and understudied caspase isoform. Starting from disulfide ligands identified in a cysteine trapping screen, we used a structure-informed covalent ligand design to produce potent, irreversible inhibitors (**3a**) and chemoproteomic probes (**13-*t***) of C6 that exhibit unprecedented selectivity over other caspase family members and high proteome selectivity. This approach and the new tools described will enable rigorous interrogation of the role of caspase-6 in developmental biology and in inflammatory and neurodegenerative diseases.

## Introduction

**Figure 1:**
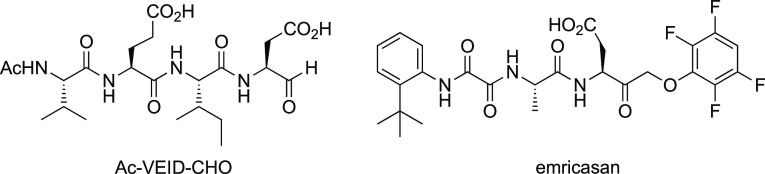
Examples of substrate-like tool compounds (left) and drug candidates (right) that engage the catalytic cysteine of caspases.

Caspases are cysteine-dependent aspartyl-specific proteases involved in a range of cellular and disease processes, ranging from apoptosis to inflammation and neurodegeneration.^1−5^ Caspase family members are broadly classified as inflammatory (C1, C4, and C5) or apoptotic caspases. The apoptotic caspases include the so-called initiator caspases (C8 and C9) that cleave executioner caspases (C3, C6, and C7) which, thus activated, go on to cleave hundreds of proteins^6^ as part of the apoptotic cell death program. The importance of caspases in various cellular processes and disease states emphasizes the potential value of isoform-selective small-molecule inhibitors and probes of caspase activity. Historically, small-molecule probes (e.g., Ac-VEID-CHO) and drug molecules (e.g., emricasan) targeting caspases have been structural mimics of caspase substrates bearing an electrophilic functionality (e.g., aldehydes or aryloxymethylketones) to engage the catalytic cysteine residue (Figure 1). Although such tools are widely available and frequently used in biomedical research, their lack of significant isoform selectivity, particularly in cellular contexts, confounds the biological or pharmacological conclusions inferred from their use.^7^ Moreover, electrophilic substrate mimics have proved challenging to develop clinically,^8,9^ likely due to insufficient selectivity and/or sub-optimal drug-like properties.

While often classified as an executioner caspase, the function of caspase-6 (C6) in physiology and disease has remained enigmatic, and it has been suggested that C6 may act upstream of the other executioner caspases.^4,10^ In addition to a putative amplifying role during apoptosis, C6 has been found to also possess nonapoptotic functions important for axon pruning and neuroinflammation.^11−14^ Finally, C6-mediated proteolysis has been implicated in the pathology of Huntington’s^15,16^ and Alzheimer’s disease,^17−19^ in neuroinflammation generally^20−22^ and in nonalcoholic steatohepatitis.^14^

**Figure 2:**
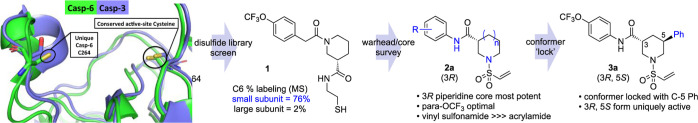
Summary of a targeted approach to the discovery of potent and selective caspase-6 inhibitors and chemical probes.

There is a clear need for potent and selective C6 inhibitors and activity-based probes to validate and exploit the role of C6 in these disease states. To produce the first truly selective probe of C6 activity in cellular contexts, we leveraged a non-conserved cysteine residue (C264) that lies on a loop at the distal end of the active site (Figure 2). While a non-catalytic cysteine is required for C8 activity^23^ and regulates the response of C9 to oxidative stress,^24^ no endogenous role for C264 in C6 regulation is known, nor has C264 to our knowledge been leveraged for inhibitor discovery previously. To achieve this, we employed disulfide trapping (tethering^25^), a mass spectrometry-based screening approach, to identify covalent probe ligands for surface-exposed/reactive cysteines in diverse proteins. The application of tethering to caspases was pioneered by Hardy and Wells,^26,27^ who showed that endogenous cysteine residues at the dimer interface of C3 and C7 could be trapped as disulfides with small molecule thiols to stabilize an inactive, zymogen-like conformation, thereby inhibiting the protease in biochemical assays. While these early thiol-based probes were not effective in the reducing environment of cells, the work inspired subsequent efforts by us and others, leading to cell-active^28^ probes of proC6 and proC8.^16,29^

## Results and Discussion

C6 possesses a non-catalytic cysteine (C264) not present in other family members and is situated on a loop in the small subunit at the distal end of the active site (Figure 2 and supplementary Figure S1). The catalytic cysteine (C163) is found on the large subunit, while two additional cysteine residues appear to be buried and thus inaccessible for disulfide formation. A disulfide tethering screen of C6 was performed employing our synthetic library of ∼1,500 diverse disulfide-bearing small molecules.^30,31^ In this technique^32^ protein-disulfide conjugates formed under reducing conditions are detected by automated HPLC/MS (supplementary Figure S2A). Notably, a large majority of screening hits labeled the small subunit, suggesting a preference for C264 over the catalytic cysteine C163 on the large subunit (supplementary Figure S2B). Moreover, labeling of the small subunit in MS experiments correlated with inhibition of C6 in biochemical assays (supplementary Figure S2C). The best C264-binding thiols contained a substituted piperidine scaffold with either a 3-amido (derived from nipecotic acid) or 4-amido linkage to the disulfide. The distal substituents varied, including substituted phenyls, 5,6-fused rings, and 6,6-fused rings (supplementary Figure S2D).

**Scheme 1:**
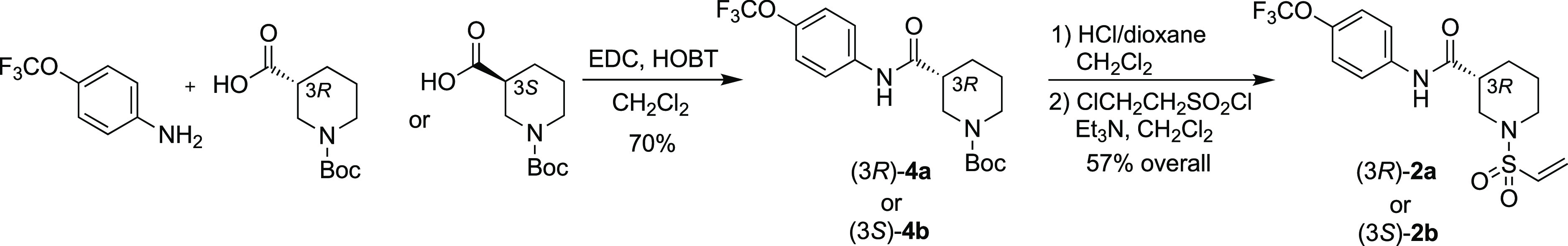
Synthesis of Enantiomers **2a** and **2b**

Among the thiol-bearing hits identified in the screen was (*S*)-nipecotic acid-derived analogue **1** bearing a distal *para*-trifluoromethoxy substituted aryl ring (Figure 2). As with the other hits identified in the screen, compound **1** labeled the small subunit almost exclusively. To derive a cell-active probe, we sought to replace the thiol side chain with a suitably positioned, electrophilic warhead. One successful foray in this vein produced analogue **2a** (R = *p*-OCF_3_), in which the nipecotic acid core was reoriented, with the piperidine nitrogen displaying a vinylsulfonamide. Analogue **2a**, having the 3*R* configuration, exhibited markedly superior C264 engagement compared to its 3*S* enantiomer **2b**, with a mass spectrometry-based dose–response-50 (DR_50_) value of 0.08 μM and a C6 inhibition IC_50_ value of 0.180 μM (Scheme 1 and Table 1), indicating that inhibition by **2a** involved both molecular recognition and reaction with C264. Notably, among several cysteine-reactive warheads explored, the vinylsulfonamide alone provided potent C6 inhibition. Also fortuitously, the *para*-trifluoromethoxyaryl ring present in the original screening hit **1** proved superior to many differently substituted aryl rings explored and was thus retained during further optimization.

**Table 1:**
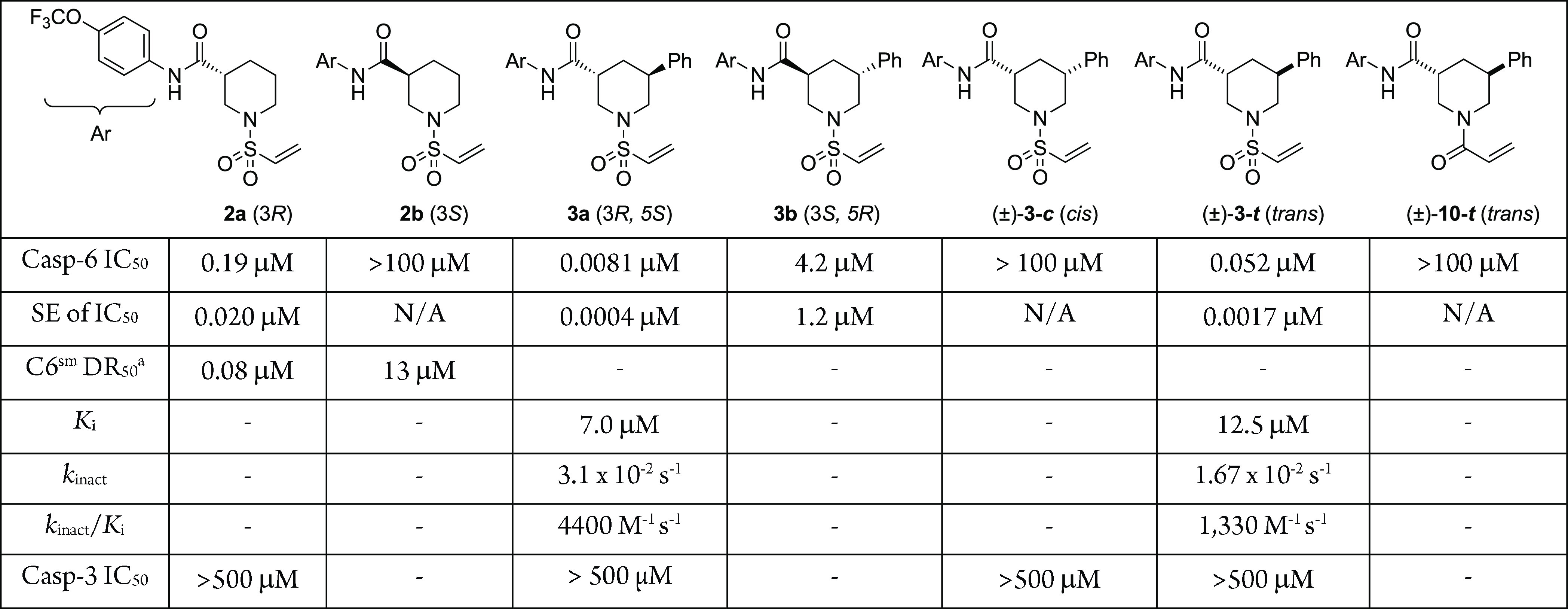
Measures of C264 Engagement, Biochemical IC_50_ Values, and Inhibition Kinetics for Caspase-6 Inhibitors

a DR_50_ is the concentration of the inhibitor, producing 50% labeling of the Casp-6 small subunit (C6^sm^), as determined by MS.

**Figure 3:**
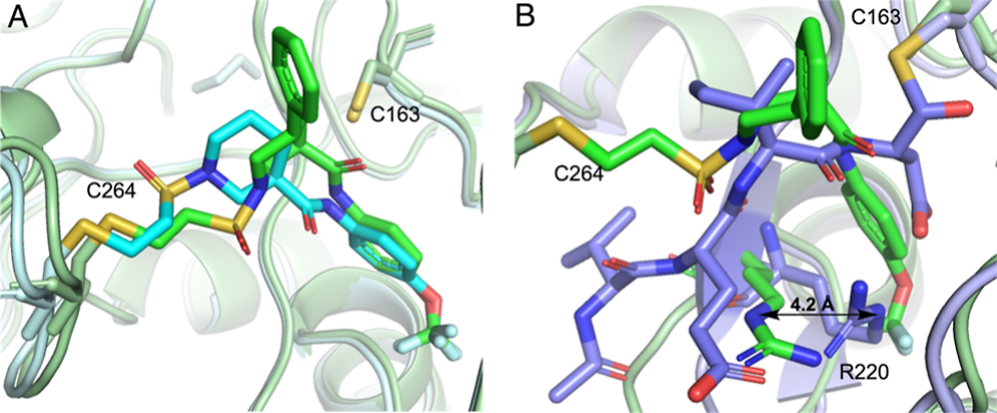
Structures of **2a**, **3a**, and Ac-VEID-CHO bound to caspase-6. (A) Superposition of crystal structures of **2a** (cyan) and **3a** (green) bound in the active site of caspase-6. The catalytic cysteine C163 is on the large subunit (at right), while inhibitor-modified C264 is on the small subunit (at left). (B) Superposition of **3ou for your a** (green, light green protein) and substrate analogue Ac-VEID-CHO (violet, light violet protein). The side chain of R220 moves 4.2 Å to accommodate the trifluoromethoxyphenyl substituent on **3a**. PDBIDs: 8EG6 (**2a**/C6); 8EG5 (**3a**/C6); 3OD5 (VEID/C6).

The crystal structure of **2a** bound to C6 confirmed the covalent modification of residue C264, whose α-carbon atom moved ∼2.4 Å toward the substrate-binding groove, compared to the structure of C6 bound to a VEID peptide substrate analogue (Figure 3). Informed by this structure, we explored new piperidine substitutions, seeking to engage additional pockets in the active site. Initial modeling suggested this might be achieved with a bulky C-5 substituent bearing a *cis* relationship to the benzamide side chain at C-3. Contrary to expectations, however, it was in fact the *trans* diastereomer (±)-**3-*t*** that realized potent C6 inhibition, while (±)-**3-*c*** was surprisingly without detectable biochemical activity (Table 1).

**Scheme 2:**
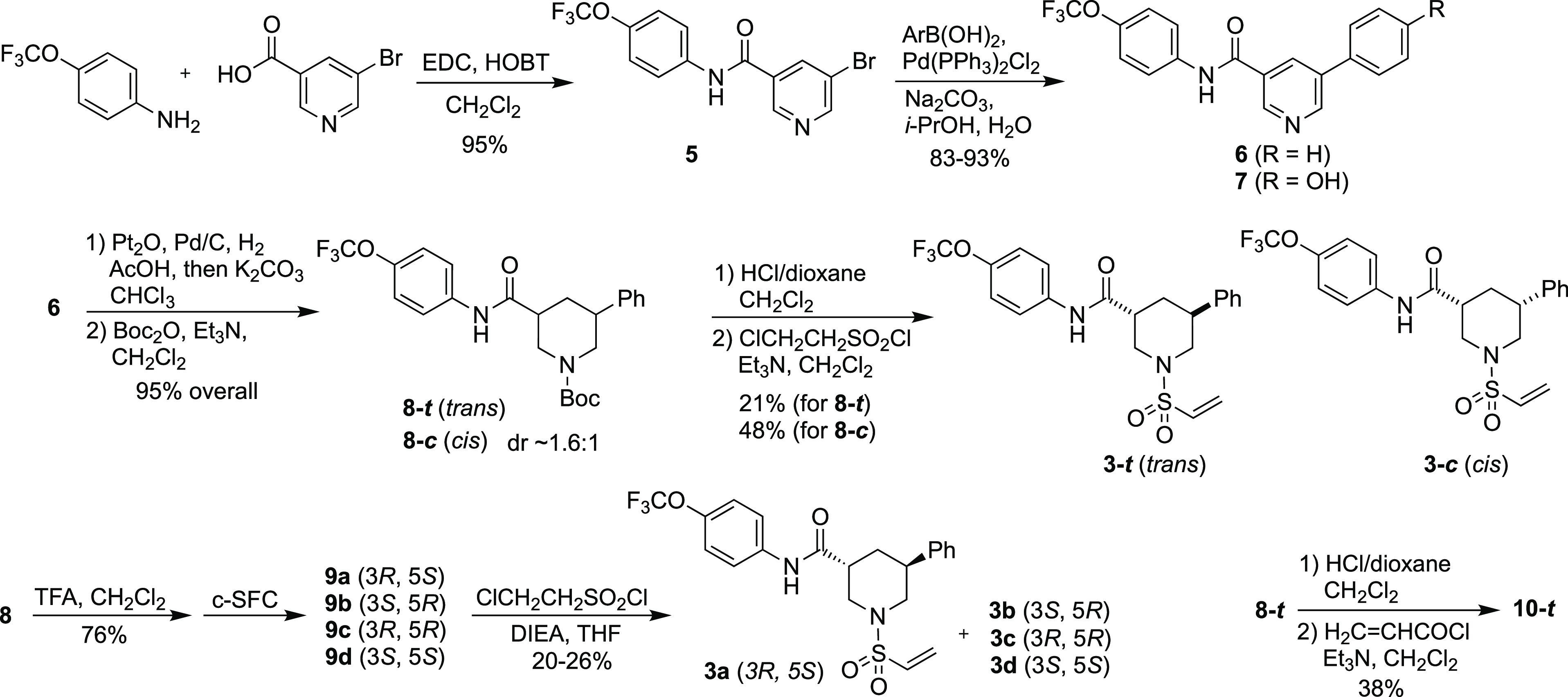
Synthesis of (±)-**3-*t*** and (±)-**3-*c***, and the Corresponding Non-Racemic Stereoisomers **3a–d** Compounds **3a–d** were prepared from the individual stereoisomers **9a–d**, which were separated by silica gel chromatography, followed by chiral-SFC. The acrylamide analogue **(±)-10-*t*** was synthesized from **(±)-8-*t,*** as shown at lower right

To assign the eutomer and distomer of (±)-**3-*t***, we separated the enantiomers of the synthetic precursor (±)-**8-*t*** (Scheme 2) and from these prepared analogues **3a** (*3R*, *5S*) and **3b** (*3S*, *5R*). When tested in a biochemical assay, enantiomer **3a** potently inhibited C6 activity with an IC_50_ value of 8 nM, while its enantiomer **3b** exhibited an IC_50_ at least 100-fold weaker (Table 1). A complex X-ray crystal structure of **3a** showed similarities and differences as compared to the binding of **2a** (Figure 3A). Both analogues engaged C264 in a covalent bond as expected, while the trifluoromethoxyaryl side chain of both analogues was buried in the S1 pocket, where the aspartic acid residue of the C6 substrate is usually bound (Figure 3A). To accommodate this large and hydrophobic ring system in S1, arginine 220, which formed hydrogen bonds with the aspartic acid in VEID peptide-type inhibitors, is moved by 4.2 Å (ε nitrogen) to create a hydrophobic cavity complementary in shape to the sidechain of **3a** (Figure 3B). The accessibility of this pocket could explain the higher hit-rate for the small-subunit vs the active-site cysteine in the primary screen. However, the location and orientation of the piperidine ring and sulfonamide function were quite different in the two analogues. The phenyl substituent present in **3a** made few, if any, contacts with the protein, but by occupying an equatorial position in the bound conformation, it energetically stabilized the required axial orientation of the trifluoromethoxy benzamide side chain of **3a,** as bound in the S1 pocket.

**Figure 4:**
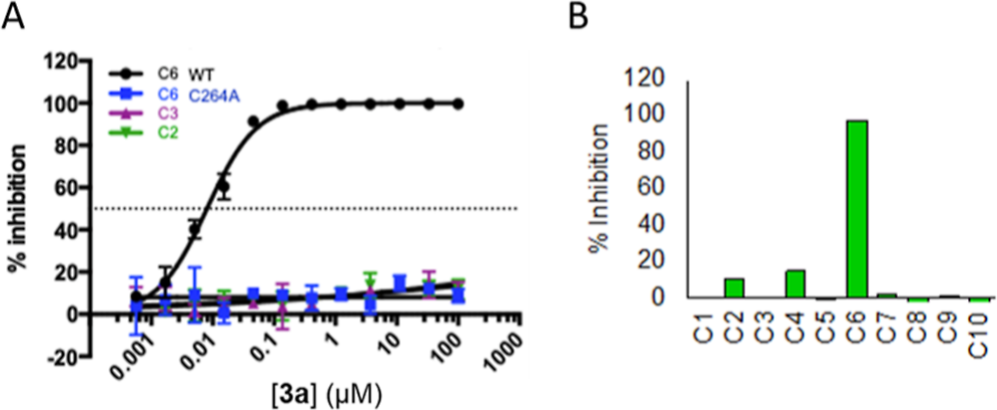
Biochemical activities of **3a**. (A) **3a** inhibits wild-type (WT) caspase-6 (C6) but not C264A C6, caspase-2 (C2), or caspase-3 (C3). Error bars represent the standard deviation of thre replicate measurements. (B) Inhibition at 10 μM **3a** of ten human caspases (C1–C10). Activity measured by the cleavage of aminofluorocoumarin-labeled peptides (see the Supporting Information for sequences). C = caspase.

To evaluate caspase isoform selectivity, we tested **3a** for the inhibition of recombinant C2, C3, and C6 WT and the C264A mutant protein in a biochemical activity assay in the presence of the fluorogenic substrate (Figure 4A). The potent low-nM inhibition of C6 WT protein by **3a** was completely lost with the C264A mutant, further establishing that **3a** engages C264. Also consistent with this requirement was the lack of activity for **3a** against either C2 or C3 (Figure 4A). Next, human caspases C1–C10 were challenged with **3a** at 10 μM (>100-fold its C6 IC_50_). In this experiment, **3a** had little, if any, effect on any of the other isoforms (Figure 4B). The potency and exquisite isoform selectivity of **3a**, together with the lack of C6 activity for its enantiomer **3b**, strongly suggested that **3a** inhibits C6 by first forming a pre-covalent complex that positioned the electrophilic vinylsulfonamide for subsequent reaction with C264. The acrylamide **10-*t***, a direct congener of **3-*t*** (Scheme 2), lacked any measurable activity against C6, further supporting the notion that inhibition was highly sensitive to proper electrophile placement and orientation.

**Figure 5:**
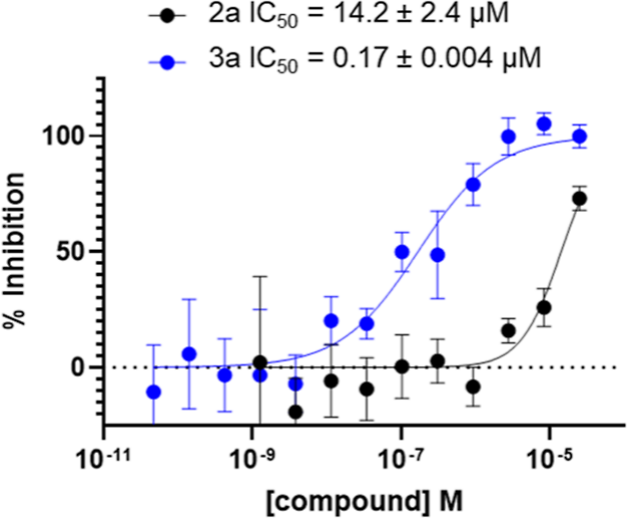
Cell-based activity of **2a** and **3a**. Inhibition of lamin A cleavage in SK-N-AS cells after staurosporine induction of caspases. Cells were treated with the indicated compound for 1 h before the addition of 10 μM staurosporine for 4 h. Error bars indicate the standard deviation from *n* = 4 replicate samples.

To better understand C6 inhibition by **3a**, we determined kinetic parameters (*k*_inact_/*K*_i_ values) of inhibition (supplementary Figure S3). These data indicated that indeed, both noncovalent and covalent interactions contributed to the binding, with a *K*_i_ value of 7.0 μM, providing an estimate of the intrinsic pre-covalent binding affinity of **3a** for C6. Moving to cellular studies, **2a** and **3a** were evaluated for their ability to inhibit cleavage of the C6-specific substrate lamin A.^33^ This assay measured lamin A cleavage under staurosporine-induced caspase activation; while staurosporine activates all executioner caspases, lamin A cleavage monitors only the activity of C6. After 1 h of preincubation with the compound and 3 h of incubation with staurosporine, lamin A cleavage was measured by in-cell western. Lamin A cleavage was inhibited by **2a** with an IC_50_ value of 14.2 ± 2.4 μM and by **3a** with an IC_50_ value of 170 ± 4 nM (Figure 5). Importantly, **2a** and **3a** inhibited lamin A cleavage in a manner that tracked well with their biochemical potencies, with **2a** roughly 50-fold less potent than **3a**. These data suggested that **2a** and **3a** function as C6-selective inhibitors in complex cellular environments containing glutathione and other cellular reductants, as well as numerous cysteine-bearing proteins.

**Figure 6:**
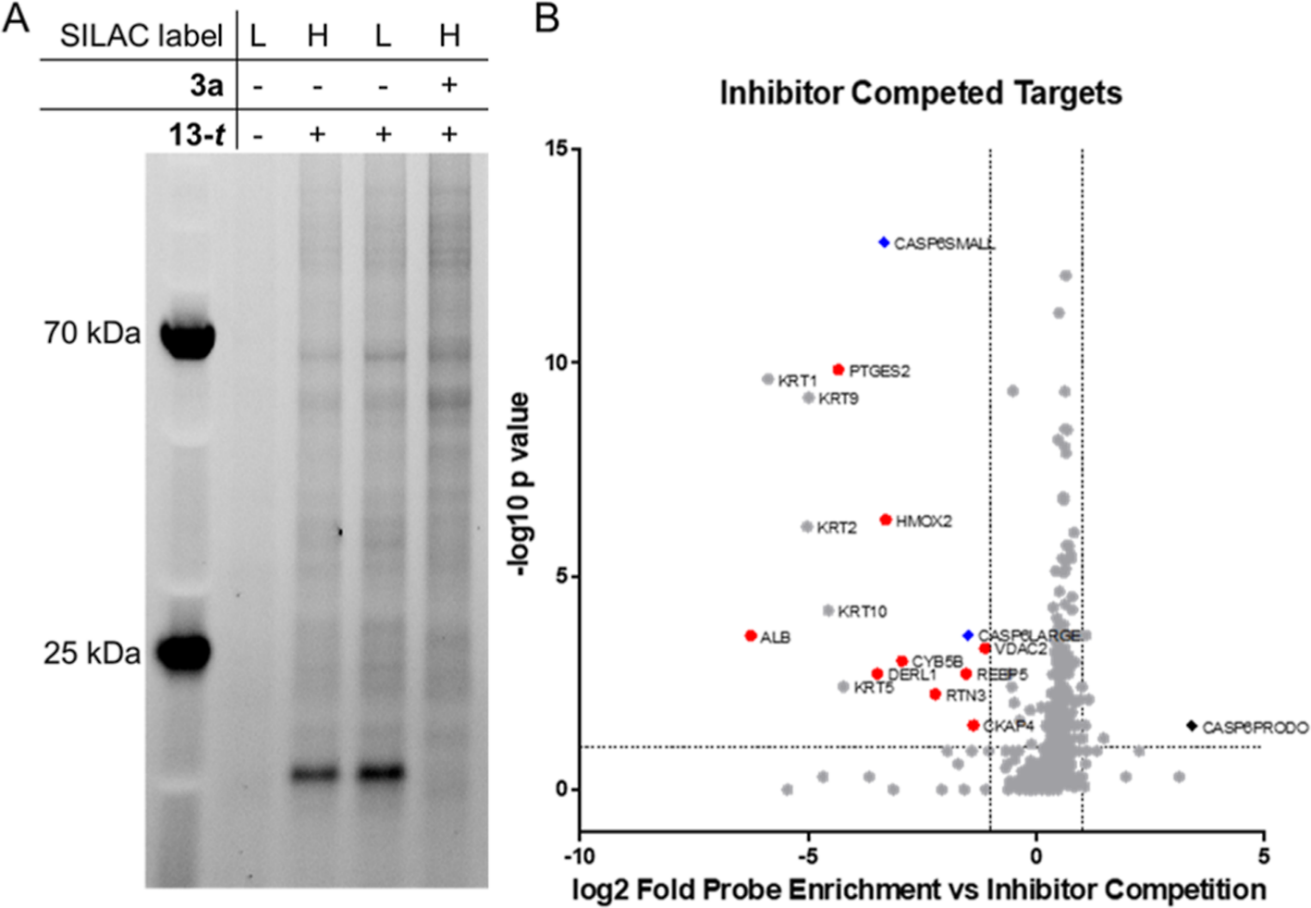
Targets of (±)-**13-*t*** (1 μM) in HEK293 cells transfected with caspase-6. (A) In gel fluorescence showing that a protein the size of the caspase-6 small subunit is a primary target of **13-*t,*** and this target is competed off by the presence of **3a**. (B) Volcano plot showing 336 total proteins identified, 141 of which were statistically significant (*p* < 0.05) targets of **13-*t*** that were competed for binding with **3a**. Red circles, competition > log 2 and *p* value <0.05; blue diamond, a small subunit of caspase-6. Keratin proteins are not highlighted because these proteins are likely present due to contamination. Data shown are representative of two independent experiments.

**Scheme 3:**
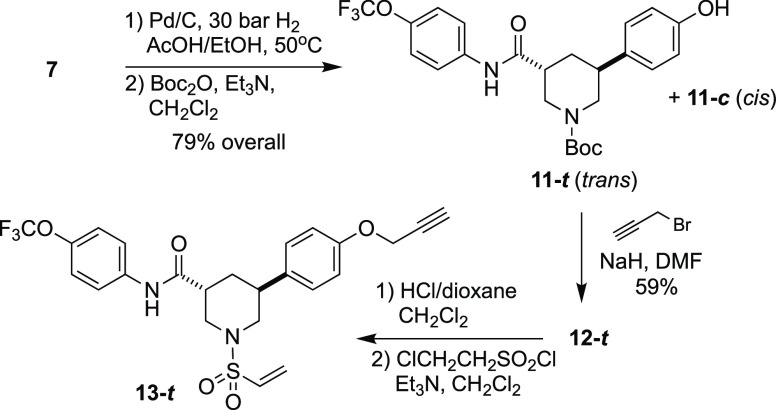
Synthesis of (±)-**13-*t,*** a Prototype Activity-Based Probe of Caspase-6, for Cellular and Chemoproteomic Studies

Having established that **3-*t***/**3a** exhibited caspase isoform selectivity and is active in cells, we sought to evaluate selectivity across the cellular proteome. We synthesized (±)-*trans*-**13** (**13-*t***), a direct congener of **3-*t*** bearing a propargyl ether substituent on the aryl side chain (Scheme 3). After confirming that **13-*t*** displayed similar potency to **3-*t*** (IC_50_ = 48 ± 2 nM, supplementary Figure S4), we evaluated the proteome-wide selectivity of this analogue in HEK293 cells using stable isotope labeling by amino acids in cell culture (SILAC) and quantitative liquid chromatography/mass spectrometry (LC/MS). Since active C6 levels were below our limit of detection for LC/MS in several cell lines (data not shown), we instead transfected HEK293 cells with an autoactivating form of caspase-6. After 24 h of transfection, 1 μM probe **13-*t*** was added to cells for 60 min, with or without 60 min of pre-treatment with 10 μM **3a** as the competitor. Cells were lysed, and heavy and light SILAC lysates were combined in a 1:1 ratio and subjected to CuAAC reaction with azido-biotin to biotinylate probe-labeled proteins for subsequent capture on streptavidin-coated beads. Bead-bound proteins were split for analysis by in-gel fluorescence and tryptic digestion on-bead for LC–MS/MS analysis. The in-gel fluorescence scan showed a dominant band around 15 kDa, consistent with the size of the caspase-6 small subunit that contains C264, the labeling of which was robustly competed by **3a** co-treatment (Figure 6A). There were also several weakly labeled bands that, in contrast to the 15 kDa band, were not significantly competed by **3a** and likely reflected weak non-specific interaction with **13-t** and/or with the surface of the streptavidin beads.

The list of LC/MS-enriched protein targets from **13-t**/**3a**-treated cells was compared to that from cells not treated with **13-*t*** or **3a**. The data revealed more than 25 proteins that were significantly (*p* < 0.05) enriched by more than log 2 fold from the **13-*t*** pulldown. The small subunit of caspase-6 was the second most highly enriched and significant protein identified in the **13-*t*** pulldown (supplementary Figure S5). Of the 25 significant interactors, only eight proteins were significantly enriched in the presence of **13-*t*** (enrichment ≥ twofold) while also being competed for by **3a**, suggesting saturable labeling by **13-*t*** (Figure 6B). Notably, the caspase-6 small subunit was the most significantly **13-*t*** enriched *and***3a**-competed protein target. None of the remaining seven putative off-target proteins were proteases; all are membrane-associated proteins, including two heme proteins (CYB5B and HMOX2) and one cysteine-dependent enzyme (PTGES2). These proteomic data indicate that **3a** exhibits meaningful, proteome-wide selectivity for caspase-6, in addition to its exquisite caspase isoform-selective inhibition.

## Conclusions

Here, we used disulfide tethering and structure-based design to produce caspase-6 inhibitors and activity-based probes of unprecedented isoform selectivity and useful proteome-wide selectivity. Compared to peptidic electrophiles currently in use, compounds **3a** and **13**-***t*** much better reflect the high bar set for chemical probes^7^ useful in biomedical research. Beyond their utility as probes, the compounds described herein represent suitable starting points for the development of potent and isoform-selective caspase-6 inhibitors as therapeutic leads. Efforts in this direction are underway and will be reported in due course.

## Supporting Information Available

A full list of proteomic targets of **13-*t*** (XLSX)
Additional experimental details, materials, and methods, including scans of ^1^H NMR spectra for **3a**–**d** and chromatograms for the separation of **9a** and **9b,** list of caspase reporter peptides, crystallography data, conservation of C264, disulfide tethering screen, Ki/kinact measurement of **3a,** caspase-6 biochemical IC_50_ data, and SILAC quantitative proteomics (PDF)
