# Supplementary material for: Engaging a Non-catalytic Cysteine Residue Drives Potent and Selective Inhibition of Caspase-6
Source: J Am Chem Soc. 2023 Apr 27;145(18):10015–21. doi: 10.1021/jacs.2c12240 (PMC10176470; doi:10.1021/jacs.2c12240)
Supplement: Supplementary file 2 — ja2c12240_si_002.pdf [file ja2c12240_si_002.pdf]

Supporting Information for

Engaging a Non-Catalytic Cysteine Residue Drives Potent and Selective Inhibition of Caspase-6

Kurt S. Van Horn,[†,‡] Dongju Wang,[†,#,‡] Daniel Medina-Cleghorn,[†] Peter S. Lee,[†] Clifford Bryant,[†] Chad Altobelli,[†] Priyadarshini Jaishankar,[†] Kevin K. Leung,[†] Raymond A. Ng,[§] Andrew J. Ambrose,[†] Yinyan Tang,[†] Michelle R. Arkin,[†,*] and Adam R. Renslo[†,*]

[†]Department of Pharmaceutical Chemistry, University of California, San Francisco, 600 16th Street, San Francisco, California 94143, United States

[#]School of Pharmaceutical Sciences, Tsinghua University, Beijing 100084, China

[§]Chempartner Corporation, 280 Utah Avenue, South San Francisco, California 94080, United States

Table of Contents

Synthetic Procedures……………….………………………….…………….….……….2

Protein expression…………………..……………………………………………15

Biochemical assays, crystallography, proteomics……………..………………………18

Supplementary Table 1: Caspase reporter peptides…..……..……...……….………20

Supplementary Table 2: Crystallography Data Table……..………..……….……..…23

Supplementary Figure 1: Conservation of C264………...…..……………….………..25

Supplementary Figure 2: Tethering Screen…………………………...………………26

Supplementary Figure 3: Ki/kinact measurement of **3a**……………………….……………27

Supplementary Figure 4: Caspase-6 biochemical IC_{50} data ……..………..……………28

Supplementary Figure 5: SILAC quantitative proteomics ……..…………………….....29

Scans of ^{1}H NMR Spectra for 3a-3d…….………………………………………..30

Supplementary Information References………………………………………………..32

Experimental

Synthetic Procedures

General information: All evaporations were carried out *in vacuo* with a rotary evaporator. Analytical samples were dried *in vacuo* (1-5 mmHg) at rt. Thin layer chromatography (TLC) was performed on silica gel plates, spots were visualized by UV light (214 and 254 nm). Purification by column and flash chromatography was carried out using silica gel (200-300 mesh). Solvent systems are reported as mixtures by volume. ^{1}H chemical shifts are reported in δ values in ppm with the deuterated solvent as the internal standard. Data are reported as follows: chemical shift, multiplicity (s = singlet, d = doublet, t = triplet, q = quartet, br = broad, m = multiplet), coupling constant (Hz), integration. LCMS spectra were obtained on an Agilent 1200 series 6110 or 6120 mass spectrometer with electrospray ionization and excepted as otherwise indicated, the general LCMS condition was as follows: Waters X Bridge C18 column (50 mm x 4.6 mm x 3.5 um), Flow Rate: 2.0 ml/min, the column temperature: 40 °C.

Materials. All chemical reagents were obtained commercially and used without further purification, unless otherwise stated. Anhydrous solvents were purchased from Sigma-Aldrich and used without further purification. Solvents used for flash column chromatography and reaction work-up procedures were purchased from either Sigma-Aldrich or Fisher Scientific. Column chromatography was performed on Silicycle Sili-prep cartridges using a Biotage Isolera Four automated flash chromatography system.

Instrumentation. NMR spectra were recorded on either a Varian INOVA 400 MHz spectrometer (with 5 mm Quad-Nuclear Z-Grad Probe), or a Bruker AvanceIII HD 400 MHz (with 5mm BBFO Z-gradient Smart Probe), calibrated to NMR solvent peaks as an internal reference. Data for ^{1}H NMR spectra are reported in terms of chemical shift (δ, ppm), multiplicity, coupling constant (Hz), and integration. Data for ^{13}C NMR spectra are reported in terms of chemical shift (δ, ppm), with multiplicity and coupling constants in the case of C–F coupling. The following abbreviations are used to denote the multiplicities; s = singlet, d = doublet, t = triplet, q = quartet, m = multiplet, br = broad, app = apparent, or combinations of these. LC-MS and compound purity were determined using

Waters Micromass ZQ 4000, equipped with a Waters 2795 Separation Module, Waters 2996 Photodiode Array Detector, and a Waters 2424 ELSD. Separations were carried out with an XBridge BEH C18, 3.5µm, 4.6 x 20 mm column, at ambient temperature (unregulated) using a mobile phase of water-methanol containing a constant 0.10 % formic acid.

***tert*-Butyl (3*R*)-3-{[4-(trifluoromethoxy)phenyl]carbamoyl}piperidine-1-carboxylate (4a)**. A combination of 0.33 mL (2.4 mmol) 4-(trifluoromethoxy)aniline, 0.52 g (2.2 mmol) (*R*)-1-*N*-(*tert*-butyloxycarbonyl)-piperidine-3-carboxylic acid, 0.48 g (2.5 mmol) 1-(3-dimethylaminopropyl)-3-ethylcarbodiimide hydrochloride and 0.033 g (0.22 mmol) 1-hydroxybenzotriazole hydrate were mixed in 8 mL methylene chloride and stirred for six hours. The solution was diluted with 50 mL water and extracted thrice with 25 mL methylene chloride. Flash chromatography using a hexanes/ethyl acetate gradient resulted in 0.56 g of the final product as a white solid in 66% yield. ^{1}H NMR (300 MHz, $CDCl_3$) δ 9.63 (s, 1H), 7.59 (d, J = 8.7 Hz, 2H), 7.04 (d, J = 8.6 Hz, 2H), 4.09 (dd, J = 13.5, 3.7 Hz, 1H), 3.90 (d, J = 13.3 Hz, 1H), 3.04 (dd, J = 13.4, 10.4 Hz, 1H), 2.81 (t, J = 12.4 Hz, 1H), 2.46 (tt, J = 9.9, 3.9 Hz, 1H), 1.98 – 1.85 (m, 1H), 1.79 (q, J = 11.8 Hz, 1H), 1.62 (s, 1H). 19F NMR (282 MHz, CDCl3) δ -58.29. ^{13}C NMR (75 MHz, $CDCl_3$) δ 172.03, 155.17, 145.15, 137.33, 121.26, 121.12, 120.56 (q, J = 256.2 Hz), 80.16, 46.04, 44.57, 43.80, 28.29, 27.82, 24.25. Mass (ESI): [M+acetonitrile+Na]$^+$ 452.2; found 452.3. R_f = 0.52 H:EA 2:1.

***tert*-Butyl (3*S*)-3-{[4-(trifluoromethoxy)phenyl]carbamoyl}-piperidine-1-carboxylate (4b)**: 0.29 mL (2.1 mmol) 4-(trifluoromethoxy)aniline, 0.45 g (1.9 mmol) (*S*)-1-*N*-(*tert*-butyloxycarbonyl)-piperidine-3-carboxylic acid, 4.2 g (2.2 mmol) 1-(3-dimethylaminopropyl)-3-ethylcarbodiimide hydrochloride and 0.029 g (0.19 mmol) 1-hydroxybenzotriazole hydrate were mixed in 7 mL methylene chloride and stirred for five hours. The solution was diluted with 50 mL water and extracted thrice with 30 mL methylene chloride. Flash chromatography using a hexanes/ethyl acetate gradient resulted in 0.52 g of the final product as a white foam in 70% yield. ^{1}H NMR (300 MHz, $CDCl_3$) δ 9.53 (s, 1H), 7.61 (d, J = 8.9 Hz, 2H), 7.08 (d, J = 8.6 Hz, 2H), 4.06 (dd, J = 13.4, 3.8 Hz, 1H), 3.88 (d, J = 13.3 Hz, 1H), 3.13 (dd, J = 13.5, 10.0 Hz, 1H), 2.90 (t, J = 12.3 Hz, 1H), 2.55 – 2.39 (m, 1H), 1.98 – 1.74 (m, 2H), 1.65 (dp, J = 12.3, 4.0 Hz, 1H), 1.44 (s, 9H). ^{19}F NMR (282 MHz, CDCl3) δ -58.23. ^{13}C NMR (75 MHz, $CDCl_3$) δ 171.94, 155.29, 145.26, 137.35, 121.41, 121.15,

120.65 (q, J = 256.3 Hz), 80.32, 46.04, 44.72, 43.87, 28.43, 27.88, 24.30. Mass (ESI): $[M+acetonitrile+Na]^+$ 452.2; found 452.3. Rf = 0.45 H:EA 1:1.

(3*R*)-1-(Ethenesulfonyl)-*N*-[4-(trifluoromethoxy)phenyl]piperidine-3-carboxamide (2a):

To a mixture of 0.56 g (1.4 mmol) *tert*-butyl (3*R*)-3-{[4-(trifluoromethoxy)phenyl]carbamoyl] piperidine-1-carboxylate (**4a**) in 15 mL methylene chloride was added 3.6 mL (14 mmol) 4 M hydrochloric acid in 1,4-dioxane. After stirring overnight the solvent was removed using a rotary evaporator. Mass (ESI): $[M+H]^+$ 289.1; found 289.2. R_f = 0.13 DCM:MeOH 9:1. To the crude (3*R*)-*N*-[4-(trifluoromethoxy)phenyl]piperidine-3-carboxamide in 30 mL of methylene chloride was added 1.0 mL (7.2 mmol) triethylamine followed by 0.76 mL (7.2 mmol) 2-chloroethanesulfonyl chloride. After stirring for four hours the solution was diluted with 100 mL water and the aqueous extracted thrice with 40 mL of methylene chloride. Flash chromatography using a hexanes/ethyl acetate gradient resulted in 0.32 g of the final product as a white solid in 58% yield over two steps. ^{1}H NMR (300 MHz, $CDCl_3$) δ 8.55 (s, 1H), 7.57 (d, J = 9.0 Hz, 2H), 7.11 (d, J = 8.7 Hz, 2H), 6.46 (dd, J = 16.6, 9.9 Hz, 1H), 6.24 (d, J = 16.6 Hz, 1H), 6.08 (d, J = 9.9 Hz, 1H), 3.78 (dd, J = 11.8, 3.7 Hz, 1H), 3.60 (d, J = 11.6 Hz, 1H), 2.94 (t, J = 11.0 Hz, 1H), 2.77 – 2.61 (m, 2H), 2.08 – 1.96 (m, 1H), 1.91 – 1.79 (m, 1H), 1.77 – 1.61 (m, 2H). ^{19}F NMR (282 MHz, $CDCl_3$) δ -58.17. ^{13}C NMR (75 MHz, $CDCl_3$) δ 171.42, 145.61, 136.80, 132.63, 128.91, 121.59, 121.40, 120.68 (q, J = 256.8 Hz), 48.04, 46.16, 43.70, 27.43, 24.15. Mass (ESI): $[M+H]^+$ 379.1; found 379.2. R_f = 0.18 H:EA 2:1.5.

(3*S*)-1-(Ethenesulfonyl)-*N*-[4-(trifluoromethoxy)phenyl] piperidine-3-carboxamide (2b). To a mixture of 0.52 g (1.3 mmol) *tert*-butyl-(3S)-3-{[4-(trifluoromethoxy)phenyl]carbamoyl] piperidine-1-carboxylate (**4b**) in 10 mL methylene chloride was added 3.3 mL (13 mmol) 4 M hydrochloric acid in 1,4-dioxane. After two hours the solvent was removed using a rotary evaporator. Mass (ESI): $[M+H]^+$ 289.1; found 289.1. R_f = 0.13 DCM:MeOH 9:1. To the crude (3*S*)-*N*-[4-(trifluoromethoxy)phenyl]piperidine-3-carboxamide in 30 mL of methylene chloride was added 0.93 mL (6.7 mmol) triethylamine followed by 0.71 mL (6.7 mmol) 2-chloroethanesulfonyl chloride. After stirring overnight the solution was diluted with 100 mL water and the aqueous extracted thrice with 50 mL of methylene chloride. Flash chromatography using a hexanes/ethyl acetate gradient resulted in 0.29 g of the final product as a white solid in 57% yield over two steps. ^{1}H NMR (300 MHz, $CDCl_3$) δ 8.60 (s, 1H), 7.56 (d, J = 9.0 Hz, 2H), 7.10 (d, J = 8.7 Hz, 2H), 6.45 (dd, J = 16.6,

9.9 Hz, 1H), 6.23 (d, J = 16.6 Hz, 1H), 6.12 – 6.02 (m, 1H), 3.78 (dd, J = 11.9, 3.7 Hz, 1H), 3.60 (d, J = 9.9 Hz, 1H), 2.91 (t, J = 11.0 Hz, 1H), 2.75 – 2.61 (m, 2H), 2.08 – 1.96 (m, 1H), 1.90 – 1.78 (m, 1H), 1.78 – 1.56 (m, 2H). ^{19}F NMR (282 MHz, $CDCl_3$) δ -58.16. ^{13}C NMR (75 MHz, $CDCl_3$) δ 171.45, 145.62, 145.59, 136.81, 132.70, 128.79, 121.53, 121.44, 120.67 (q, J = 256.8 Hz), 48.02, 46.12, 43.69, 27.41, 24.15. Mass (ESI): $[M+H]^+$ 379.1; found 379.0. R_f = 0.47 H:EA 1:1.

5-Bromo-*N*-[4-(trifluoromethoxy)phenyl]pyridine-3-carboxamide (5). A mixture of 2.36 mL (17.3 mmol) 4-(trifluoromethoxy)aniline, 2.91 g (14.4 mmol) 5-bromonicotinic acid, 2.9 g (15.1 mmol) 1-(3-dimethylaminopropyl)-3-ethylcarbodiimide hydrochloride and 0.025 g (0.16 mmol) 1-hydroxybenzotriazole hydrate were mixed in 60 mL methylene chloride and stirred for six hours. The solution was diluted with 100 mL water, 100 mL brine, 100 mL saturated aqueous ammonium chloride and 200 mL acetone. The organic layer was washed once more with 100 mL water, 100 mL brine, and 100 mL saturated aqueous ammonium chloride and washed twice with 200 mL brine. 5.3 g (95%) of the final product was collected as a white solid that was used without further purification in the next step. ^{1}H NMR (300 MHz, $CDCl_3$) δ 8.97 (s, 1H), 8.83 (s, 1H), 8.33 (d, J = 2.4 Hz, 1H), 8.10 (s, 1H), 7.71 – 7.61 (m, 2H), 7.24 (d, J = 10.5 Hz, 2H).^{19}F NMR (282 MHz, $CDCl_3$) δ -58.09. ^{13}C NMR (75 MHz, acetone-d_6) δ 164.31, 154.70, 148.83, 146.64, 139.52, 139.24, 133.73, 123.29, 123.18, 122.24 (q, J = 254.6 Hz), 121.81. Mass (ESI): $[M+H]^+$ 361.0; found 361.0, 363.1. R_f = 0.38 D:M 24:1.

5-Phenyl-*N*-[4-(trifluoromethoxy)phenyl]pyridine-3-carboxamide (6). A mixture of 1.97 g (5.46 mmol) 5-bromo-*N*-[4-(trifluoromethoxy)phenyl]pyridine-3-carboxamide (**5**), 1 g (8.2 mmol) phenylboronic acid, 2.89 g (27.3 mmol) sodium carbonate and 0.10 g (0.15 mmol) dichlorobis(triphenylphosphine)palladium were suspended in 30 mL isopropanol and 7 mL water. Nitrogen was bubbled through the mixture for 5 minutes and the reaction was heated at 80 ºC. After two hours the reaction was cooled, diluted with 100 mL water, and extracted twice with 80 mL ethyl acetate. The combined organic layer was washed twice with 100 mL brine and the solution concentrated. Flash chromatography using a hexanes/ethyl acetate gradient resulted in 1.63 g of the final product as a tan solid in 83% yield. ^{1}H NMR (300 MHz, Methanol-d_4) δ 9.04 (s, 1H), 8.97 (s, 1H), 8.56 (s, 1H), 7.84 (d, J = 8.8 Hz, 2H), 7.74 (d, J = 7.1 Hz, 2H), 7.59 – 7.40 (m, 3H), 7.28 (d, J = 8.5 Hz, 2H). ^{19}F NMR (282 MHz, CD_3OD) δ -59.68. ^{13}C NMR (75 MHz, CD_3OD) δ 166.23, 151.03,

148.05, 146.78, 138.74, 138.27, 137.75, 135.25, 132.36, 130.39, 129.87, 128.27, 123.39, 122.62. Mass (ESI): $[M+H]^+$ 359.1; found 359.1. $R_f = 0.57$ H:EA 1:1.

tert*-Butyl 3-phenyl-5-{[4-(trifluoromethoxy)phenyl]carbamoyl}piperidine-1-carboxylate (8)**. A mixture of 1.63 g (4.55 mmol) 5-phenyl-*N*-[4-(trifluoromethoxy)phenyl]pyridine-3-carboxamide (**6**), 0.11 g (0.455 mmol) platinum (IV) oxide hydrate and 0.095 g (0.09 mmol) 10% palladium on carbon were stirred in 20 mL acetic acid with an attached hydrogen balloon. After the starting material was consumed as judged by TLC (generally 48 – 72 hours), the reaction was filtered through celite and rinsed with chloroform. The solvent was removed by rotary evaporation, the crude dissolved in 20 mL chloroform and stirred with 1 g potassium carbonate. After one hour the solution was filtered through a glass frit, the solids rinsed with chloroform and the solvent evaporated. Mass (ESI): $[M+H]^+$ 365.1; found 365.3. $R_f = 0.33$, 0.21 D:M 9:1. To the crude compound in 16 mL chloroform was added 1.26 mL (9.1 mmol) triethylamine followed by 1.56 mL (6.8 mmol) di-*tert*-butyl dicarbonate. After stirring overnight the solution was diluted with 100 mL water and extracted three times with 50 mL methylene chloride. Flash chromatography using a hexanes/ethyl acetate gradient resulted in 0.86 g of *trans* product **8-*t, along with 0.52 g of the pure *cis* product **8-*c***, and 0.63 g of a **8-*t*/8-*c*** mixture as white solids in an overall combined 95% yield over two steps. ^{1}H NMR (**8-*c***) (300 MHz, $CDCl_3$) δ 8.60 (s, 1H), 7.56 (d, $J = 8.9$ Hz, 2H), 7.35 – 7.28 (m, 2H), 7.25 – 7.18 (m, 3H), 7.13 (d, $J = 8.6$ Hz, 2H), 4.38 (d, $J = 13.3$ Hz, 1H), 4.32 – 4.11 (m, 1H), 2.99 (t, $J = 12.4$ Hz, 1H), 2.91 – 2.74 (m, 1H), 2.74 – 2.51 (m, 2H), 2.24 – 2.00 (m, 2H), 1.47 (s, 9H). ^{19}F NMR (**8-*c***) (282 MHz, $CDCl_3$) δ -58.12. ^{1}H NMR (**8-*t***) (300 MHz, $CDCl_3$) δ 9.31 (s, 1H), 7.76 (d, $J = 8.6$ Hz, 2H), 7.37 – 7.29 (m, 2H), 7.29 – 7.21 (m, 3H), 7.18 (d, $J = 8.6$ Hz, 2H), 4.48 (d, $J = 14.6$ Hz, 1H), 4.12 (dd, $J = 13.2$, 3.9 Hz, 1H), 3.28 (dd, $J = 14.6$, 3.5 Hz, 1H), 3.15 (dd, $J = 13.2$, 10.3 Hz, 1H), 2.92 (s, 1H), 2.81 (t, $J = 4.0$ Hz, 1H), 2.68 (d, $J = 11.9$ Hz, 1H), 1.95 (td, $J = 12.7$, 4.7 Hz, 1H), 1.48 (s, 9H). ^{19}F NMR (**8-*t***) (282 MHz, $CDCl_3$) δ -58.04. ^{13}C NMR (**8-*t***) (75 MHz, $CDCl_3$) δ 171.12, 155.50, 145.19, 145.16, 142.36, 137.32, 128.58, 127.04, 126.81, 121.36, 121.17, 120.60 (q, $J = 256.4$ Hz), 80.88, 77.59, 77.17, 76.74, 50.33, 44.30, 41.52, 39.02, 33.94, 28.34. Mass (ESI): $[M+acetonitrile+Na]^+$ 528.2; found 528.3. R_f (**8-*c***) = 0.66 H:EA 2:1. R_f (**8-*t***) = 0.56 H:EA 2:1.

***Trans*-1-(ethenesulfonyl)-5-phenyl-*N*-[4-(trifluoromethoxy)phenyl]piperidine-3-carboxamide (3-*t*)**. To a mixture of 0.25 g (0.55 mmol) *tert*-butyl *trans*-3-phenyl-5-{[4-(trifluoromethoxy)phenyl]

carbamoyl}piperidine-1-carboxylate (**8-*t***) in 5 mL methylene chloride was added 1.35 mL (5.5 mmol) 4 M hydrochloric acid in 1,4-dioxane. After stirring overnight the solvent was removed using a rotary evaporator to afford *trans*-5-phenyl-*N*-[4-(trifluoromethoxy)phenyl]piperidine-3-carboxamide as the HCl salt. Mass (ESI): $[M+H]^+$ 365.1; found 365.1. R_f = 0.30 DCM:MeOH 9:1. To this crude intermediate in 5 mL of methylene chloride was added 0.8 mL (5.7 mmol) triethylamine followed by 0.12 mL (1.1 mmol) 2-chloroethanesulfonyl chloride. After one hour the solution was diluted with 20 mL water and the aqueous extracted thrice with 10 mL of methylene chloride. Flash chromatography using a hexanes/ethyl acetate gradient resulted in 0.057 g of the final product as a white solid in 21% yield over two steps. ^{1}H NMR (400 MHz, $CDCl_3$) δ 8.52 (s, 1H), 7.70 (d, *J* = 8.8 Hz, 2H), 7.31-7.36 (m, 2H), 7.24-7.19 (m, 5H), 6.49 (dd, *J* = 16.5, 9.9 Hz, 1H), 6.33 (d, *J* = 16.6 Hz, 1H), 6.14 (d, *J* = 9.8 Hz, 1H), 4.03 (d, *J* = 13.3 Hz, 1H), 3.85 (dd, *J* = 11.6, 4.0 Hz, 1H), 3.08 (dd, *J* = 13.0, 3.2 Hz, 1H), 3.02 (dt, *J* = 11.1, 4.0 Hz, 1H), 2.91 (p, *J* = 3.9 Hz, 1H), 2.85 (t, *J* = 11.0 Hz, 1H), 2.62 (dt, *J* = 12.8, 3.4 Hz, 1H), 1.93 (ddd, *J* = 13.4, 11.7, 5.0 Hz, 1H). ^{19}F NMR (282 MHz, $CDCl_3$) δ -58.11. ^{13}C NMR (100 MHz, $CDCl_3$) δ 170.52, 145.66, 145.64, 141.23, 136.83, 132.25, 129.75, 128.90, 127.41, 127.26, 121.74, 121.37, 120.71 (q, *J* = 256.8 Hz), 51.77, 46.73, 40.86, 38.86, 33.52. Mass (ESI): $[M+H]^+$ 455.1; found 455.1. R_f = 0.51 H:EA 1:1.

Cis*-1-(ethenesulfonyl)-5-phenyl-*N*-[4-(trifluoromethoxy)phenyl]piperidine-3-carboxamide (3-*c*).** To 0.032 g (0.07 mmol) *tert*-butyl *cis*-3-phenyl-5-{[4-(trifluoromethoxy)phenyl]carbamoyl} piperidine-1-carboxylate (**8-*c) in 2 mL methylene chloride was added 0.18 mL (0.7 mmol) 4 M hydrochloric acid in 1,4-dioxane. After 2 hours the solvent was removed using a rotary evaporator to afford *cis*-5-phenyl-*N*-[4-(trifluoromethoxy)phenyl]piperidine-3-carboxamide as the HCl salt. Mass (ESI): $[M+H]^+$ 365.1; found 365.4. R_f = 0.34 DCM:MeOH 9:1. To this crude intermediate in 3 mL of methylene chloride was added 0.1 mL (0.7 mmol) triethylamine followed by 0.015 mL (0.14 mmol) 2-chloroethanesulfonyl chloride. After 2 hours the solution was diluted with 20 mL water and the aqueous phase extracted thrice with 10 mL of methylene chloride. Flash chromatography using a hexanes/ethyl acetate gradient resulted in 0.015 g of the final product as a white solid in 48% yield over two steps. ^{1}H NMR (300 MHz, $CDCl_3$) δ 8.20 (s, 1H), 7.58 (d, *J* = 9.0 Hz, 2H), 7.38 – 7.24 (m, 3H), 7.24 – 7.18 (m, 2H), 7.15 (d, *J* = 8.5 Hz, 2H), 6.49 (dd, *J* = 16.6, 9.9 Hz, 1H), 6.28 (d, *J* = 16.6 Hz, 1H), 6.11 (d, *J* = 9.9 Hz, 1H), 4.06 (d, *J* = 8.4 Hz, 1H), 3.89 (dd, *J* = 11.8, 4.0 Hz, 1H), 3.11 – 2.83 (m, 3H), 2.64 (t, *J* = 11.7 Hz, 1H), 2.31 (d, *J* = 13.3 Hz, 1H), 2.07 – 1.90 (m, 1H). ^{19}F NMR

(282 MHz, $CDCl_3$) δ -58.12. ^{13}C NMR (75 MHz, $CDCl_3$) δ 171.13, 145.74, 141.33, 136.72, 132.78, 129.05, 128.99, 127.56, 127.22, 121.69, 121.40, 120.73 (q, J = 256.9), 52.00, 47.95, 44.54, 41.88, 34.77. Mass (ESI): $[M+H]^+$ 455.1; found 455.2. R_f = 0.77 H:EA 1:1.

Trans-5-phenyl-1-(prop-2-enoyl)-*N*-[4-(trifluoromethoxy)phenyl]piperidine-3-carboxamide (10-*t*). To 0.080 g (0.17 mmol) *tert*-butyl *trans*-3-phenyl-5-{[4-(trifluoromethoxy)phenyl]carbamoyl}piperidine-1-carboxylate (**8-*t***) in 3 mL methylene chloride was added 0.45 mL (1.8 mmol) 4 M hydrochloric acid in 1,4-dioxane. After five hours the solvent was removed using a rotary evaporator resulting in 68 mg of *trans*-5-phenyl-*N*-[4-(trifluoromethoxy)phenyl]piperidine-3-carboxamide as the HCl salt. R_f = 0.22 DCM:MeOH 9:1. To 15 mg (0.037 mmol) of this crude intermediate in 1 mL of methylene chloride was added 0.03 mL (0.2 mmol) triethylamine followed by 6 µL (0.075 mmol) acryloyl chloride. After two hours the solution was diluted with 10 mL water and the aqueous extracted thrice with 10 mL of methylene chloride. Preparative HPLC resulted in 0.006 g of the final product as a white solid in 38% yield over two steps. ^1H NMR (300 MHz, $CDCl_3$) δ 9.77 (s, 1H), 7.76 (d, J = 9.0 Hz, 2H), 7.37 – 7.27 (m, 3H), 7.25 – 7.13 (m, 4H), 6.57 (dd, J = 16.7, 10.2 Hz, 1H), 6.41 (dd, J = 16.8, 2.0 Hz, 1H), 5.80 (dd, J = 10.2, 2.0 Hz, 1H), 5.16 (dd, J = 14.7, 2.4 Hz, 1H), 4.11 (d, J = 12.7 Hz, 1H), 3.28 (t, J = 12.3 Hz, 1H), 3.11 (dd, J = 14.9, 3.5 Hz, 1H), 2.99 (d, J = 2.8 Hz, 1H), 2.86 (d, J = 13.7 Hz, 1H), 2.71 (tt, J = 11.9, 3.9 Hz, 1H), 2.00 (td, J = 13.0, 5.2 Hz, 1H). ^{19}F NMR (282 MHz, $CDCl_3$) δ -58.07. ^{13}C NMR (100 MHz, $CDCl_3$) δ 170.20, 166.99, 145.48, 141.71, 137.55, 129.64, 129.00, 127.46, 127.12, 126.96, 121.60, 121.49, 120.80 (q, J = 256.5 Hz), 52.93, 42.89, 42.41, 40.72, 34.60. Mass (ESI): $[M+H]^+$ 419.2; found 419.0. R_f = 0.38 H:EA 1:1.

5-(4-Hydroxyphenyl)-*N*-[4-(trifluoromethoxy)phenyl]pyridine-3-carboxamide (7). A mixture of 1.04 g (2.88 mmol) 5-bromo-*N*-[4-(trifluoromethoxy)phenyl]pyridine-3-carboxamide (**5**), 0.52 g (3.8 mmol) 4-hydroxyphenylboronic acid, 1.53 g (14.4 mmol) sodium carbonate, and 0.055 g (0.08 mmol) dichlorobis(triphenylphosphine)palladium were suspended in 10 mL isopropanol and 2.5 mL water. Nitrogen was bubbled through the mixture for 5 minutes and the reaction was heated at 80 ºC. After two hours the reaction was cooled, diluted with 50 mL water, and extracted twice with 30 mL ethyl acetate. The combined organic layer was washed twice with 40 mL brine and the solution concentrated on silica gel. Flash chromatography using a hexanes/ethyl acetate gradient afforded in

1.0 g of the final product as a white solid in 93% yield. ^{1}H NMR (300 MHz, Methanol-d_4) δ 8.91 (d, J = 2.1 Hz, 1H), 8.84 (d, J = 2.2 Hz, 1H), 8.43 (t, J = 2.1 Hz, 1H), 7.86 – 7.76 (m, 2H), 7.57 – 7.50 (m, 2H), 7.24 (d, J = 8.7 Hz, 2H), 6.95 – 6.87 (m, 2H). ^{19}F NMR (282 MHz, Methanol-d_4) δ -59.61. ^{13}C NMR (75 MHz, Methanol-d_4) δ 166.34, 159.65, 150.34, 146.91, 146.73, 138.67, 138.17, 134.39, 132.16, 129.42, 128.57, 123.62, 122.56, 121.93 (q, J = 255.3 Hz), 120.24, 117.18. Mass (ESI): $[M+H]^+$ 375.1; found 375.1. R_f = 0.57 H:EA 1:1.

tert*-Butyl-3-(4-hydroxyphenyl)-5-{[4-(trifluoromethoxy)phenyl]carbamoyl}piperidine-1-carboxylate (11-*t* and 11-*c*)**. A solution of 1.0 g (2.67 mmol) 5-(4-hydroxyphenyl)-*N*-[4-(trifluoromethoxy)phenyl]pyridine-3-carboxamide (**7**) in 40 mL of 45% acetic acid in ethanol was placed on an H-cube flow hydrogenator system with a Pd/C cartridge and continuously cycled at 50 ºC and 30 bar hydrogen overnight. The solvent was then removed by rotary evaporation to afford the crude piperidine product, which was used without further purification. Mass (ESI): $[M+H]^+$ 381.1; found 381.3. R_f = 0.17, 0.10 DCM:MeOH 9:1. To the crude piperidine intermediate in 25 mL methylene chloride was added 1.0 mL (7.2 mmol) triethylamine followed by 0.66 mL (2.9 mmol) di-*tert*-butyl dicarbonate. After stirring two hours the solution was diluted with 50 mL water and extracted three time with 25 mL methylene chloride. The crude compound was absorbed on silica gel and purified with flash chromatography using a hexanes/ethyl acetate gradient to afford 0.4 g of the *cis* product **11-*c, along with 0.4 g of the *trans* product **11-*t***, and 0.2 g of a **11-*c*/11-*t*** mixture as white solids in combined 79% yield over two steps. ^{1}H NMR (**11-*c***) (300 MHz, CD_3OD) δ 7.61 (d, J = 8.5 Hz, 2H), 7.13 (d, J = 8.6 Hz, 2H), 7.02 (d, J = 8.2 Hz, 2H), 6.72 (d, J = 8.2 Hz, 2H), 4.57 (s, 1H), 4.25 (d, J = 12.2 Hz, 1H), 4.14 – 3.95 (m, 1H), 2.91 (s, 1H), 2.72 – 2.53 (m, 3H), 2.10 (d, J = 12.2 Hz, 1H), 1.97 – 1.78 (m, 1H), 1.42 (s, 9H). ^{19}F NMR (**11-*c***) (282 MHz, CD_3OD) δ -59.47. ^{13}C NMR (**11-*c***) (75 MHz, CD_3OD) δ 174.06, 157.21, 156.44, 146.43, 138.62, 134.54, 128.93, 122.58, 122.37, 121.85 (q, J = 255.6 Hz), 116.52, 81.51, 49.85, 47.03, 45.43, 42.36, 36.41, 28.69. ^{1}H NMR (**11-*t***) (300 MHz, $CDCl_3$) δ 9.23 (s, 1H), 8.05 (s, 1H), 7.71 (d, J = 8.8 Hz, 2H), 7.16 (d, J = 8.6 Hz, 2H), 6.98 (d, J = 8.4 Hz, 2H), 6.79 (d, J = 8.4 Hz, 2H), 4.38 (d, J = 13.7 Hz, 1H), 4.02 (d, J = 10.3 Hz, 1H), 3.29 (d, J = 12.1 Hz, 1H), 3.20 – 3.04 (m, 1H), 2.80 (s, 2H), 2.58 (d, J = 10.5 Hz, 1H), 1.96 – 1.77 (m, 1H), 1.46 (s, 9H). ^{19}F NMR (**11-*t***) (282 MHz, $CDCl_3$) δ -59.09. ^{13}C NMR (**11-*t***) (75 MHz, $CDCl_3$) δ 171.70, 155.74, 155.28, 145.40, 136.88, 133.50, 128.04, 122.24, 121.41, 115.63, 81.25,

50.52, 44.36, 41.56, 38.16, 34.01, 28.35. Mass (ESI): [M+acetonitrile+Na]$^+$ 544.2; found 544.4. R_f (S17) = 0.62 H:EA 1:1. R_f (S18) = 0.50 H:EA 1:1.

tert*-Butyl trans-3-[4-(prop-2-yn-1-yloxy)phenyl]-5-{[4-(trifluoromethoxy)phenyl]carbamoyl} piperidine-1-carboxylate (12-*t*).** To 0.40 g (0.83 mmol) *tert*-butyl *trans*-3-(4-hydroxyphenyl)-5-{[4-(trifluoromethoxy)phenyl]carbamoyl}piperidine-1-carboxylate (**11-*t) in 4 mL *N*,*N*-dimethylformamide was added 0.1 g (2.5 mmol) 60% sodium hydride in mineral oil. After stirring five minutes 0.135 mL (1.25 mmol) 80% propargyl bromide was added. After stirring one hour the reaction was diluted with 100 mL water and extracted three times with 20 mL methylene chloride. Flash chromatography using a hexanes/ethyl acetate gradient resulted in a yellow oil contaiting 0.24 g of the desired product in 56% yield. ^{1}H NMR (300 MHz, $CDCl_3$) δ 9.37 (s, 1H), 7.55 (d, *J* = 8.7 Hz, 2H), 7.02 (d, *J* = 8.2 Hz, 2H), 6.96 (d, *J* = 8.6 Hz, 2H), 6.74 (d, *J* = 8.3 Hz, 2H), 4.49 (d, *J* = 2.4 Hz, 2H), 4.01 (d, *J* = 14.2 Hz, 1H), 3.72 (dd, *J* = 13.0, 4.1 Hz, 1H), 3.35 – 3.21 (m, 1H), 3.11 (dd, *J* = 13.3, 8.9 Hz, 1H), 2.62 (s, 2H), 2.44 (s, 1H), 2.33 (s, 1H), 1.79 (ddt, *J* = 13.7, 10.4, 5.4 Hz, 1H), 1.25 (s, 9H). ^{19}F NMR (282 MHz, $CDCl_3$) δ -58.25. ^{13}C NMR (75 MHz, $CDCl_3$) δ 171.30, 156.15, 154.86, 144.60, 137.49, 135.58, 127.94, 121.01, 120.75, 120.31 (q, *J* = 256.2 Hz), 114.77, 80.05, 78.53, 75.33, 55.69, 49.53, 44.64, 40.60, 37.39, 33.54, 28.08. Mass (ESI): [M+acetonitrile+Na]$^+$ 582.2; found 582.4. R_f = 0.75 H:EA 1:1.

Trans-1-(ethenesulfonyl)-5-[4-(prop-2-yn-1-yloxy)phenyl]-*N*-[4-(trifluoromethoxy)phenyl]piperidine-3-carboxamide (13-*t*). To 0.24 g (0.46 mmol) *tert*-butyl trans-3-[4-(prop-2-yn-1-yloxy)phenyl]-5-{[4-(trifluoromethoxy)phenyl]carbamoyl}piperidine-1-carboxylate (**12-*t***) in 20 mL methylene chloride was added 1.7 mL (6.8 mmol) 4 M hydrochloric acid in 1,4-dioxane. After stirring overnight the solvent was removed using a rotary evaporator to afford *trans*-5-[4-(prop-2-yn-1-yloxy)phenyl]-*N*-[4-(trifluoromethoxy)phenyl]piperidine-3-carboxamide as the HCl salt. Mass (ESI): [M+H]$^+$ 419.2; found 419.1. R_f = 0.24 DCM:MeOH 9:1. To this crude intermediate in 20 mL of methylene chloride was added 0.64 mL (4.6 mmol) triethylamine followed by 0.10 mL (0.91 mmol) 2-chloroethanesulfonyl chloride. After one hour the solution was diluted with 20 mL water and the aqueous extracted thrice with 10 mL of methylene chloride. Flash chromatography using a hexanes/ethyl acetate gradient resulted in 0.059 g of the final product as a white solid in 26% yield over two steps. ^{1}H NMR (300 MHz, $CDCl_3$) δ 8.52 (s, 1H), 7.74 – 7.64 (m,

2H), 7.19 (d, J = 3.1 Hz, 2H), 7.16 (d, J = 2.2 Hz, 2H), 6.97 – 6.87 (m, 2H), 6.48 (dd, J = 16.6, 9.8 Hz, 1H), 6.31 (d, J = 16.6 Hz, 1H), 6.13 (d, J = 10.1 Hz, 1H), 4.66 (d, J = 2.3 Hz, 2H), 3.97 (d, J = 12.8 Hz, 1H), 3.78 (dd, J = 11.5, 3.9 Hz, 1H), 3.08 (dd, J = 13.0, 3.3 Hz, 1H), 2.99 (td, J = 10.7, 5.2 Hz, 1H), 2.91 – 2.77 (m, 2H), 2.61 – 2.53 (m, 1H), 2.52 (t, J = 2.3 Hz, 1H), 1.88 (ddd, J = 13.7, 11.4, 5.0 Hz, 1H). ^{19}F NMR (282 MHz, $CDCl_3$) δ -58.09. ^{13}C NMR (75 MHz, $CDCl_3$) δ 170.48, 156.99, 145.69, 136.82, 134.31, 132.24, 129.77, 128.30, 121.77, 121.36, 115.48, 78.75, 75.66, 56.14, 51.98, 46.70, 40.92, 38.14, 33.73. Mass (ESI): $[M+H]^+$ 509.1; found 509.1. R_f = 0.34 H:EA 1:1.

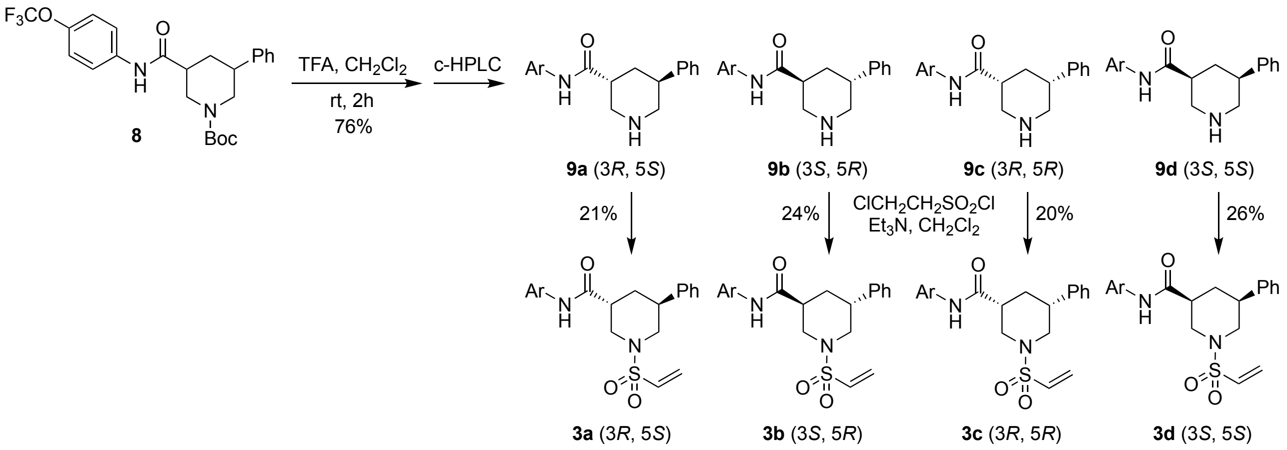

Scheme S1. Use of intermediate **8** and to prepare **3a-d** via chiral HPLC separation of intermediate **9**. The relative stereochemistry of **3a/b** as *trans* and **3c/d** as *cis* were assigned by comparison to the racemic mixtures **3-*t*** and **3-*c*** prepared using the route described in the main text and as detailed above.

Synthesis of 5-phenyl-*N*-(4-(trifluoromethoxy)phenyl)piperidine-3-carboxamide (9a–d). To a solution of **8** (4.2 g, 9.05 mmol) in dichloromethane (10 mL) was added TFA (10 mL), and the mixture was stirred at room temperature for 2 h. The solvent was removed *in vacuo*. The residue was dissolved in dichloromethane (60 mL), and then the mixture was neutralized with saturated $NaHCO_3$ (aq.), washed with brine (60 mL), dried over anhydrous Na_2SO_4, filtered and concentrated *in vacuo* to afford crude **9** (2.5 g, yield: 76%) as a white solid. This material was first purified by silica gel column chromatography in ethyl acetate/hexanes to acheive separation of the *trans* and *cis* isomers as racemates. The racemic mixtures were further separated by chiral-supercritical fluid chromatography (c-SFC) on CHIRALPAK AD-3 (4.6 x 100mm) with co-solvent of methanol (with 0.2% 7M NH_3) to provide **9a** (0.10 g, yield: 3%) as a white solid, **9b** (0.10 g, yield: 3%) as a white solid, **9c** (0.80 g, yield: 24%) as a white solid, **9d** (0.80 g, yield: 24%) as a white solid. Further details for the chiral-SFC conditions are provided in the chromatogram of **9a** and **9b** below.

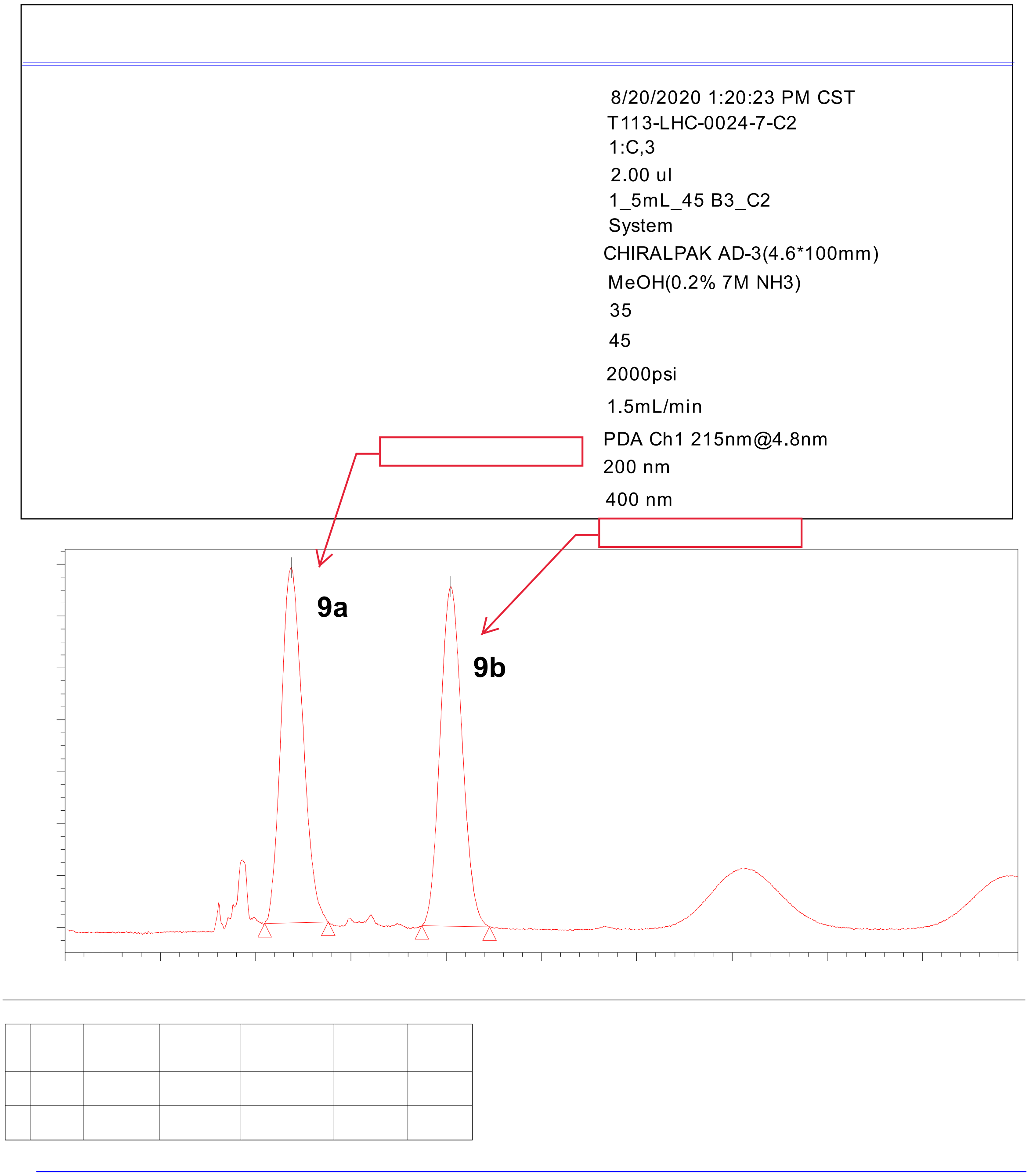

Synthesis of (3*R*, 5*S*)-1-(ethenesulfonyl)-5-phenyl-*N*-[4-(trifluoromethoxy)phenyl]piperidine - 3-carboxamide (3a). To a solution of **9a** (100 mg, 0.27 mmol) and *N,N*-diisopropylethylamine (105 mg, 0.81 mmol) in tetrahydrofuran (10 mL) was added 2-chloroethanesulfonyl chloride (89.1

mg, 0.55 mmol) under ice-water bath. The mixture was allowed to warm to room temperature and stirred for 1 h. Water (30 mL) and ethyl acetate (30 mL) was added. The organic layer was separated. The aqueous layer was extracted with ethyl acetate (2 x 30 mL). The combined organic layers were washed with brine (30 mL), dried over anhydrous Na_2SO_4, filtered and concentrated *in vacuo* to give crude product which was purified by pre-HPLC and chiral-HPLC to give **3a** (26 mg, yield: 21%) as a white solid**.**

LC-MS (Agilent LCMS 1200-6120, Column: Waters X-Bridge C18 (50 mm*4.6 mm*3.5 μm); Column Temperature: 40 °C; Flow Rate: 2.0 mL/min; Mobile Phase: from 95% [water + 10 mM NH_4HCO_3] and 5% [CH_3CN] to 0% [water + 10 mM NH_4HCO_3] and 100% [CH_3CN] in 1.6 min, then under this condition for 1.4 min, finally changed to 95% [water + 10 mM NH_4HCO_3] and 5% [CH_3CN] in 0.1 min and under this condition for 0.7 min). Purity is 97.92%, Rt =2.257 min; MS Calcd.:454.12; MS Found: 455.3 $[M+H]^+$.

HPLC (Agilent HPLC 1200, Column: Waters X-Bridge C18 (150 mm*4.6 mm*3.5 μm); Column Temperature: 40 °C; Flow Rate: 1.0 mL/min; Mobile Phase: from 95% [water + 10 mM NH_4HCO_3] and 5% [CH_3CN] to 0% [water + 10 mM NH_4HCO_3] and 100% [CH_3CN] in 10 min, then under this condition for 5 min, finally changed to 95% [water + 10 mM NH_4HCO_3] and 5% [CH_3CN] in 0.1 min and under this condition for 5 min). Purity is 100.0%, Rt =10.965 min.

The ^{1}H NMR spectra of this material was consistent with that of racemic *trans*-**3** (**3-*t***) prepared by the route described above and in the main text. The assignment of absolute stereochemistry is based on the crystal structure obtained from **3-*t*** (PDB:8EG5) which revealed the (3*R*, 5*S*) form to be bound (i.e., **3a**) and by caspase-6 IC_{50} values which revealed this material to be the active stereoisomer, and thus **3a**.

^{1}H NMR (400 MHz, $CDCl_3$) δ 8.49 (s, 1H), 7.70-7.72 (m, 2H), 7.31-7.34 (m, 2H), 7.19-7.27 (m, 5H), 6.46-6.53 (m, 1H), 6.32-6.36 (m, 1H), 6.15 (d, *J* = 9.6 Hz, 1H) 4.05-4.08 (m, 1H), 3.87-3.91 (m, 1H), 2.99-3.09 (m, 2H), 2.90-2.93 (m, 1H), 2.82 (t, *J* = 10.8 Hz, 1H), 2.63-2.66 (m, 1H), 1.88-1.96 (m, 1H).

Synthesis of (3*S*, 5*R*)-1-(ethenesulfonyl)-5-phenyl-*N*-[4-(trifluoromethoxy)phenyl]piperidine -3-carboxamide (3b). To a solution of **9b** (100 mg, 0.27 mmol) and *N,N*-diisopropylethylamine

(105 mg, 0.81 mmol) in tetrahydrofuran (10 mL) was added 2-chloroethanesulfonyl chloride (89.1 mg, 0.55 mmol) under ice-water bath. The mixture was allowed to warm to room temperature and stirred for 1 h. Water (30 mL) and ethyl acetate (30 mL) was added. The organic layer was separated. The aqueous layer was extracted with ethyl acetate (2 x 30 mL). The combined organic layers were washed with brine (30 mL), dried over anhydrous Na_2SO_4, filtered and concentrated *in vacuo* to give crude product which was purified by pre-HPLC and chiral-HPLC to give **3b** (30 mg, yield: 24%) as a white solid.

LC-MS (Agilent LCMS 1200-6120, Column: Waters X-Bridge C18 (50 mm*4.6 mm*3.5 μm); Column Temperature: 40 °C; Flow Rate: 2.0 mL/min; Mobile Phase: from 95% [water + 10 mM NH_4HCO_3] and 5% [CH_3CN] to 0% [water + 10 mM NH_4HCO_3] and 100% [CH_3CN] in 1.6 min, then under this condition for 1.4 min, finally changed to 95% [water + 10 mM NH_4HCO_3] and 5% [CH_3CN] in 0.1 min and under this condition for 0.7 min). Purity is 100.0%, Rt =2.260 min; MS Calcd.:454.12; MS Found: 455.3 $[M+H]^+$.

HPLC (Agilent HPLC 1200, Column: Waters X-Bridge C18 (150 mm*4.6 mm*3.5 μm); Column Temperature: 40 °C; Flow Rate: 1.0 mL/min; Mobile Phase: from 95% [water + 10 mM NH_4HCO_3] and 5% [CH_3CN] to 0% [water + 10 mM NH_4HCO_3] and 100% [CH_3CN] in 10 min, then under this condition for 5 min, finally changed to 95% [water + 10 mM NH_4HCO_3] and 5% [CH_3CN] in 0.1 min and under this condition for 5 min). Purity is 100.0%, Rt =10.978 min.

The ^1H NMR of this material was consistent with that of *trans*-**3** (**3-*t***) prepared by the route described above and in the main text. The assignment of absolute stereochemistry is based on the crystal structure obtained from **3-*t*** and the caspase-6 IC_{50} values which revealed this material to be the less active enantiomer, and thus **3b**.

^1H NMR (400 MHz, $CDCl_3$) δ 8.48 (s, 1H), 7.70-7.72 (m, 2H), 7.31-7.34 (m, 2H), 7.19-7.28 (m, 5H), 6.46-6.53 (m, 1H), 6.31-6.36 (m, 1H) , 6.15 (d, J = 9.6 Hz, 1H) 4.05-4.08 (m, 1H), 3.87-3.90 (m, 1H), 2.99-3.09 (m, 2H), 2.90-2.94 (m, 1H), 2.82 (t, J = 11.0 Hz, 1H), 2.63-2.66 (m, 1H), 1.88-1.96 (m, 1H).

Synthesis of (3*R*, 5*R*)-1-(ethenesulfonyl)-5-phenyl-*N*-[4-(trifluoromethoxy)phenyl]piperidine -3-carboxamide (3c). To a solution of **9c** (100 mg, 0.27 mmol), and *N,N*-diisopropylethylamine (105

mg, 0.81 mmol) in tetrahydrofuran (10.0 mL) was added 2-chloroethanesulfonyl chloride (89.1 mg, 0.55 mmol) under ice-water bath. The mixture was allowed to warm to room temperature and stirred for 1 h. Water (30 mL) and ethyl acetate (30 mL) was added. The organic layer was separated. The aqueous layer was extracted with ethyl acetate (2 x 30 mL). The combined organic layers were washed with brine (30 mL), dried over anhydrous Na_2SO_4, filtered and concentrated *in vacuo* to give crude product which was purified by pre-HPLC and chiral-HPLC to give **3c** (25 mg, yield: 20%) as a white solid**.**

LC-MS (Agilent LCMS 1200-6120, Column: Waters X-Bridge C18 (50 mm*4.6 mm*3.5 μm); Column Temperature: 40 °C; Flow Rate: 2.0 mL/min; Mobile Phase: from 95% [water + 10 mM NH_4HCO_3] and 5% [CH_3CN] to 0% [water + 10 mM NH_4HCO_3] and 100% [CH_3CN] in 1.6 min, then under this condition for 1.4 min, finally changed to 95% [water + 10 mM NH_4HCO_3] and 5% [CH_3CN] in 0.1 min and under this condition for 0.7 min). Purity is 100.0%, Rt =2.276 min; MS Calcd.:454.12; MS Found: 455.3 $[M+H]^+$.

HPLC (Agilent HPLC 1200, Column: Waters X-Bridge C18 (150 mm*4.6 mm*3.5 μm); Column Temperature: 40 °C; Flow Rate: 1.0 mL/min; Mobile Phase: from 95% [water + 10 mM NH_4HCO_3] and 5% [CH_3CN] to 0% [water + 10 mM NH_4HCO_3] and 100% [CH_3CN] in 10 min, then under this condition for 5 min, finally changed to 95% [water + 10 mM NH_4HCO_3] and 5% [CH_3CN] in 0.1 min and under this condition for 5 min). Purity is 100.0%, Rt =11.154min.

The ^{1}H NMR of this material was consistent with that of *cis*-**3** (**3-*c***) prepared by the route described in the main text and thus confirms the relative stereochemistry. The assignment of absolute stereochemistry for **3c** and **3d** are arbitrary.

^{1}H NMR (400 MHz, $CDCl_3$) δ 7.98 (s, 1H), 7.48-7.51 (m, 2H), 7.24-7.28 (m, 2H), 7.15-7.21 (m, 3H), 7.09 (d, *J* = 8.8 Hz, 2H), 6.38-6.44 (m, 1H), 6.18-6.22 (m, 1H), 6.02 (d, *J* = 10.0 Hz, 1H), 3.96-3.98 (m, 1H), 3.83-3.85 (m, 1H), 2.94-3.00 (m, 1H), 2.86 (t, *J* = 11.2 Hz, 1H), 2.75-2.81 (m, 1H), 2.57 (t, *J* = 11.6 Hz, 1H), 2.22-2.25 (m, 1H), 1.88-1.98 (m, 1H).

Synthesis of (3*S*, 5*S*)-1-(ethenesulfonyl)-5-phenyl-N-[4-(trifluoromethoxy)phenyl]piperidine -3-carboxamide (3d). To a solution of **9d** (100 mg, 0.27 mmol) and *N,N*-diisopropylethylamine (105 mg, 0.81 mmol) in tetrahydrofuran (10.0 mL) was added 2-chloroethanesulfonyl chloride (89.1 mg,

0.55 mmol) under ice-water bath. The mixture was allowed to warm to room temperature and stirred for 1 h. Water (30 mL) and ethyl acetate (30 mL) was added. The organic layer was separated. The aqueous layer was extracted with ethyl acetate (2 x 30 mL). The combined organic layers were washed with brine (30 mL), dried over anhydrous Na_2SO_4, filtered and concentrated *in vacuo* to give crude product which was purified by pre-HPLC and chiral-HPLC to give **9d** (32 mg, yield: 26%) as a white solid**.**

LC-MS (Agilent LCMS 1200-6120, Column: Waters X-Bridge C18 (50 mm*4.6 mm*3.5 μm); Column Temperature: 40 °C; Flow Rate: 2.0 mL/min; Mobile Phase: from 95% [water + 10 mM NH_4HCO_3] and 5% [CH_3CN] to 0% [water + 10 mM NH_4HCO_3] and 100% [CH_3CN] in 1.6 min, then under this condition for 1.4 min, finally changed to 95% [water + 10 mM NH_4HCO_3] and 5% [CH_3CN] in 0.1 min and under this condition for 0.7 min). Purity is 96.50%, Rt =2.281 min; MS Calcd.:454.12; MS Found: 455.2 $[M+H]^+$.

HPLC (Agilent HPLC 1200, Column: Waters X-Bridge C18 (150 mm*4.6 mm*3.5 μm); Column Temperature: 40 °C; Flow Rate: 1.0 mL/min; Mobile Phase: from 95% [water + 10 mM NH_4HCO_3] and 5% [CH_3CN] to 0% [water + 10 mM NH_4HCO_3] and 100% [CH_3CN] in 10 min, then under this condition for 5 min, finally changed to 95% [water + 10 mM NH_4HCO_3] and 5% [CH_3CN] in 0.1 min and under this condition for 5 min). Purity is 98.45%, Rt =11.164 min.

The ^1H NMR of this material was consistent with that of *cis*-**3** (**3-*c***) prepared by the route described in the main text and thus confirms the relative stereochemistry. The assignment of absolute stereochemistry for **3c** and **3d** are arbitrary.

^1H NMR (400 MHz, $CDCl_3$) δ 7.78 (s, 1H), 7.54-7.58 (m, 2H), 7.32-7.35 (m, 2H), 7.23-7.29 (m, 3H), 7.18 (d, J = 8.4 Hz, 2H), 6.43-6.50 (m, 1H), 6.24-6.28 (m, 1H), 6.08 (d, J = 10.0 Hz, 1H), 4.02-4.05 (m, 1H), 3.88-3.92 (m, 1H), 2.99-3.03 (m, 1H), 2.92 (t, J = 11.6 Hz, 1H), 2.78-2.84 (m, 1H), 2.63 (t, J = 11.8 Hz, 1H), 2.27-2.30 (m, 1H), 1.97-2.07 (m, 1H).

Caspase-6 protein expression: Human caspase-6 was expressed as the mature enzyme using a split construct cloned into a pETDUET vector with two T7 promoters (Novagen). Residues 30-179 (LS) and 194-293-Leu-Glu-His_6 (SS) from caspase-6 cDNA (Origene) were cloned using the following primers:

C6duet_LS_forward: CGGCGCGCCTGCAGGTCGACatgtttgatccggcagaaaagtac
C6duet_LS_reverse: CTTTCTGTTCGACTTAAGCATTAatctactacatccaaaggcatgac.
C6duet_SS_forward: GTTAAGTATAAGAAGGAGATATACATgcagcctccgttacacgctgcctg
C6duet_SS_reverse: CGCAGCAGCGGTTTCTTTACCAGATCAttaattagattttggaaagaaatg

The pETDUET vector transfected into *E.coli* strain Rosetta 2 pLysS DE3 (Novagen). Cells were grown from a single colony in 6L of 2xYT supplemented with carbenicillin at 37 °C to an OD of ~ 0.5 and were subsequently cooled to 16 °C before overnight induction with 1 mM IPTG. Cells were harvested by centrifugation, resuspended in 100 mM NaCl, 100 mM Tris, pH 8.0, 1 mM βME and lysed by sonication. The lysate was clarified by centrifugation at 20,000xg for 20 minutes and the supernatant was loaded onto a 1 mL HisTrap FF column (GE Healthcare), washed with 80% buffer A (100 mM NaCl, 100 mM Tris pH 8.0), 20% buffer B (100 mM NaCl, 100 mM Tris pH 8.0, 200 mM imidazole), and eluted with 100% B. The product was then diluted with 40 mL of 20 mM Tris pH 8, loaded onto a 1 mL HiTrap Q HP column (GE Healthcare), and eluted in a gradient of 0-50% 20 mM Tris pH 8.0, 1 M NaCl against 20 mM Tris pH 8.0 over 30 column volumes. Caspase-6 typically eluted with ~200 mM NaCl. Fractions were collected, concentrated by ultrafiltration and assayed for purity by denaturing gel electrophoresis and by mass spectrometry (LCT Premier; Waters). Expression of caspase-6 by this approach yielded 3-10 mg of pure protein per 6 liters of culture.

Disulfide tethering assay: The primary disulfide tethering screen was performed by incubating caspase-6 with individual compounds in a 384-well plate format. A custom library of 1,563 disulfide-containing fragments of the UCSF Small Molecule Discovery Center (SMDC), synthesized as previously reported,[1,2] was available as 50 mM stock solutions in DMSO. Caspase-6 was diluted to 500 nM in buffer (20 mM Tris, 500 µM β-mercaptoethanol (βME), pH 8.0) and plated in 384-well plates (30 µL/well). 30 nL of each fragment was pinned from the library master plates into the protein samples using a Biomek FX (Beckman) to give a final fragment concentration of 100 µM. The reaction mixtures were incubated for 6 hours at RT before being subjected to LC/MS (UPLC, LCT Premier Time of Flight mass spectrometer, Waters). Data were processed using Waters software and analyzed for % labeling according to the formula: %Tethering = (intensity of protein-compound conjugate mass)/(unconjugated protein + protein-βME conjugate + protein-compound conjugate) x

100. Raw screening data are available from HiTS (https://hits.ucsf.edu/assay/summary/1175) after registering for a public account.

Hits from the primary screen were subjected to disulfide tethering dose-response analysis at 10 concentrations ranging from 100 μM to 190 nM in a buffer consisting of 50 mM HEPES pH 8.0, 100 mM NaCl, 1 mM EDTA, 500 μM βME and 3.33% DMSO. Fragments were incubated with Caspase-6 for 1 hour before being subjected to LC/MS.

Caspase-6 enzyme assay: The in vitro enzymatic caspase-6 assays utilize synthetic tetrapeptide substrates labeled with either Rhodamine110 (R110) or 7-Amino-4-(trifluoromethyl)coumarin (AFC) at the P1 aspartic acid (Asp) residue, as described.[3] Briefly, assays were performed in 384-well plates consisting of caspase-6, VEID-Rh110 or Ac-VEID-AFC and indicated concentration of inhibitor or DMSO in assay buffer (50 mM HEPES pH 8.0, 100 mM NaCl, 1 mM EDTA, and 500 μM βME). All inhibitors were serially diluted in 100% DMSO prior to dilution in assay buffer and transfer to assay plate, for a final DMSO concentration of 1%. Compound and protein were preincubated for 15 minutes prior to addition of substrate. The reaction plate was incubated at room temperature for 30 minutes and then read on an Envision (Perkin Elmer) fluorescent plate reader at excitation/emission wavelengths of 485/535 nm (R110) or 395/495 nm (AFC). Data were processed to generate dose-response curves and analyzed using a nonlinear curve fit with variable slope in Prism software (Graphpad). For measurements of kinact/Ki, reactions were monitored without preincubation of compound; fluorescence was read at short time intervals to generate a rate curve, from which kobs was calculated using Prism.

Caspase selectivity assays: Caspase selectivity assays were contracted to Eurofins/Panlabs. Recombinant caspase was preincubated with 10 μM **3a** (1% DMSO) for 15 min before addition of a caspase reporter substrate (Table S1) for the prescribed time. Fluorescence was then read with λ_{ex} = 400-405 nm, λ_{em} = 510-535 nm, and normalized to fluorescence in the absence of added **3a**.

Supplementary Table 1. Fluorescent reporter peptides for each caspase assay

Caspase	Fluorogenic peptide
**1**	Z-YVAD-AFC
**2**	Z-VDVAD-AFC
**3**	Ac-DEVD-AMC
**4**	Ac-LEVD-AFC
**5**	Ac-WEHD-AFC
**6**	Ac-VEID-AMC
**7**	Ac-DEVD-AMC
**8**	Ac-IETD-AMC
**9**	Ac-LEHD-AFC
**10**	Ac-LEHD-AFC

Co-crystallization of caspase-6 with inhibitors: Complexes of caspase-6 with compounds were generated by mixing 1mg/ml active caspase-6 with a 5-fold molar excess of compound **2a** or **3a** for 2 hours. The reaction mixture was desalted via spin column, concentrated, and crystallized by hanging drop method with 1.7 µL drops, as follows: For **2a** and **3a**, the crystals grew from a drop containing 0.5 µl of the complex (6mg/ml), 1 µl of well solution (0.1 M TRIS pH 8.5, 12% w/v PEG 6000) and 0.2 µl seed at 18℃. Crystals were cryopreserved in well solution plus 20% ethylene glycol.

Diffraction data was collected at SPRING-8 BEAMLINE BL45XU. The data were indexed, integrated and scaled using XDS. The structure was solved by molecular replacement using the Caspase-6 structure as the search model (PDB-ID 3P4U). The initial FoFc electron density maps show unambiguous density bound compound in the active sites. The compound was fit to the density and the model was subjected to iterative cycles of refinement and rebuilding using Refmac[4] and Coot[5] (Table S2).

Lamin cleavage assay: Lamin A has been shown to be a caspase-6-specific substrate[6–8] and therefore serves as a useful target-engagement assay in cells. We used the procedure of Mintz, et al, with the following modifications: SK-N-AS cells were seeded at 10,000 cells/well in black clear bottom 384-

well plates coated with poly-D-lysine and allowed to adhere overnight. Wells were treated with varying doses of casapase-6 inhibitor, from 5 μM – 0.1 nM in 2-fold dilutions. After one hour, cells were treated with 10 μM staurosporine to induce apoptosis and activation of multiple caspases. 3 hours after staurosporine addition, cells were fixed and permeabilized before in-cell westerns were performed, as described.[9]

SILAC labeling and alkyne probe treatment: HEK293T cells were seeded into 8x 15 cm plates at a density of 500,000 cells/plate. 4 of these plates were grown in light SILAC DMEM and 4 were grown in heavy SILAC DMEM all containing 10% FBS and 4.5g/L glucose. The next day, all 8 plates were transfected with a pCMV6-Entry plasmid coding for caspase-6-myc-DDK lacking the prodomain. After 24 hours, media was replaced with fresh SILAC growth media containing 4.5 g/L glucose and 3% FBS. 24 hours later the plates were treated with probe and inhibitor for 1 hours as follows: 2 of the light plates were treated with vehicle, 2 light plates and 2 heavy plates treated with 1 μM **13-*t*** for 60 minutes, and 2 heavy plates treated with 10 μM **3a** for 60 minutes then 1 μM **13-*t*** for 60 minutes. After treatment, media was removed, and cells were washed with 15 mL PBS for 5 minutes. Cells were then harvested by scraping in cold PBS and lysed in the presence of Roche complete protease inhibitors. Lysates were clarified by centrifugation at 10,000xg for 10 minutes and the protein in the supernatant was quantified by BCA assay.

Proteomics assay: 1 mg of each lysate was diluted to 2mg/ml in PBS with Roche complete protease inhibitors. To this was added 0.1 mM biotin-azide, 1 mM tris(2-carboxyethyl)phosphine (TCEP), 0.1 mM Tris((1-benzyl-4-triazolyl)methyl)amine (TBTA) ligand, and 1 mM $CuSO_4$ in a final volume of 0.5 mL. The reaction was incubated at room temperature for 1 hour and was vortexed every 15 minutes. After 1 hour, proteins are precipitated and are pelleted by centrifuging at 16,000x*g* for 4 min at 4 °C. The supernatants were discarded, and the pellets were solubilized with 0.5 mL cold

methanol. At this point, the light and heavy samples were combined (light vehicle combined with heavy **13-*t*** treated and light **13-*t*** treated was combined with heavy **13-*t* + 3a** treated) and the samples were pelleted by centrifugation as above. The cold methanol wash was then repeated, and the washed pellets were resuspended in 1 mL of 1.2% SDS and sonicated until clear. The samples were heat denatured at 80-90 °C for 5 minutes and any insoluble particles were removed by centrifugation at 6,500xg for 5 minutes. The 1 mL supernatant was transferred to a 15 mL conical tubes containing 5 mL of PBS and 150 µL of washed neutravidin beads were added. The samples were incubated for 2 hours at 4 °C while rotating, at which point the samples were warmed to resolubilize any precipitated SDS. The beads were pelleted by centrifugation at 1400xg for 3 min and washed for 10 minutes with the following solutions: 2x 1% NP-40, 2x PBS + 1 M NaCl, and 2x 50 mM ammonium bicarbonate (ABC) + 2 M urea. The beads were then transferred to a 1.5 mL microcentrifuge tube using 2x250 µL 100 mM Tris pH 8.0 + 4 M Guanidium hydrochloride (final volume 500 µL). The samples were then reduced by adding Dithiothreitol (DTT) to a final concentration of 50 mM and incubating at 65 °C for 20 minutes. The samples were cooled to room temperature and alkylated with 20 mM iodoacetamide at 37 °C for 30 minutes with agitation. The beads were then washed 2x with 950 µL 50 mM ABC and pellet by centrifugation at 1,400xg for 3 minutes. The bead bound proteins were then trypsinized by adding 2 µg of trypsin to each sample in 50 mM ABC + 2 M Urea and incubating overnight at 37 °C. Reactions were spun down and supernatants transferred to new 1.5 microfuge tube, the pellets were washed 2x 200 µL into the new tubes with 50 mM ABC + 2 M Urea. Samples were then acidified with 10 % trifluoroacetic acid (TFA) to a final concentration of 0.5%. The samples were then applied to activated C18 SOLA HRP columns equilibrated with 0.1% TFA and the flowthrough was collected in a clean tube. The column was then washed twice with 0.5 mL 0.1% TFA and once with 0.1% formic acid (FA) in 2% acetonitrile (ACN). The peptides were then eluted

into a clean microcentrifuge tube with 100 µL of 0.1% FA in ACN and again with 150 µL of the same solution. Solvent was removed from the eluate using a Genevac and the dry peptides were resuspended in 0.1% FA in 2% ACN. Peptides in these solutions were quantified by absorbance at 280 nM and diluted to 2 µg/mL stock solutions. These stock solutions were diluted in 1:10 for MS working solutions that were injected on a Thermo Q-Exactive LC/MS.

Mass spectrometry

LC-MS/MS was performed using an UltiMate 3000 UHPLC system (Thermo) with pre-packed 0.75mm x 150mm Acclaim Pepmap C18 reversed phase columns (2µm pore size, Thermo) coupled to a Q Exactive Plus (Thermo Fisher Scientific) mass spectrometer. For each sample, 1 µg of material was injected and separated using a linear gradient of 3-40% solvent B (solvent A: 0.1% formic acid, solvent B: 80% acetonitrile, 0.1% formic acid) over 83 mins at 200 µL/min. Data-dependent acquisition was performed using a top 15 method (dynamic exclusion 20 seconds; exclusion of peptides with an unassigned charge or charge of 1). Full spectra with a resolution of 70,000 were gathered in MS1 using an AGC target of 3e6, maximum injection time of 100 ms, and scan range of 350 - 1500 m/z. Centroided data from MS2 scans were collected at a resolution of 17,500 with an AGC target of 5e4 and maximum injection time of 150 milliseconds. The normalized collision energy was set at 27 and an isolation window of 1.7 m/z with an isolation offset of 0 m/z was used.

Data analysis/Statistics

SILAC proteomics data were analyzed using an in-house pipeline as previously described, with slight modification.[10,11] A peptide search against the human proteome (SwissProt database, August 3, 2017 release, 20,218 entries) was performed using ProteinProspector v5.13.1. Enzyme specificity was set to trypsin with up to two missed cleavages. Cysteine carbamidomethyl was set

as the only fixed modification; methionine oxidation, N-terminal glutamate to pyroglutamate, and lysine/arginine SILAC labels were set as variable modifications. For the search, peptide mass tolerance was set at 6 ppm, fragment ion mass tolerance was 0.4 Da, and peptide identification was filtered by peptide score of 0.0005 in ProteinProspector, resulting in a false discovery rate (FDR) of <1% calculated using the number of decoy peptides in the SwissProt database. Skyline[12] software was used to perform quantitative analysis of SILAC ratios. For datasets collected in forward and reverse SILAC mode, spectral libraries of experiments were analyzed simultaneously to allow MS1 peaks without an explicit peptide ID to be quantified using an aligned peptide retention time. The Skyline report was exported and a previously reported custom R script was used for ratiometric analysis.[10,11] Briefly, identifications with an isotope dot product <0.8 were removed, as were proteins with fewer than two identified peptides. SILAC ratios were centered on a mean of zero, presented as median $\log_2$ enrichment values, and significance determined using a Mann-Whitney test of peptide ratios for all peptides associated with the identified protein. Data was visualized using R.

Supplementary Table 2. Crystallography data collection and refinement statistics.

	8EG6 (Caspase-6 + 2a)	8EG5 (Caspase-6 + 3a)
Wavelength	1.00000	1.00000
Resolution range	49.33 - 1.82 (1.885 - 1.82)	49.44 - 2.14 (2.216 - 2.14)
Space group	P 1 21 1	P 1 21 1
Unit cell	84.58 60.73 101.7 90 91.039 90	84.77 60.88 101.26 90 91.412 90
Total reflections	628670 (64104)	388315 (38594)
Unique reflections	92726 (9218)	57293 (5699)
Multiplicity	6.8 (7.0)	6.8 (6.8)
Completeness (%)	99.92 (99.91)	99.86 (99.95)
Mean I/sigma(I)	13.21 (2.17)	10.65 (2.14)
Wilson B-factor	23.13	28.67
R-merge	0.0992 (0.8538)	0.1492 (0.9068)
R-meas	0.1075 (0.9221)	0.1618 (0.9826)
R-pim	0.04094 (0.3454)	0.06188 (0.3752)
CC1/2	0.998 (0.776)	0.996 (0.709)
CC*	1 (0.935)	0.999 (0.911)
Reflections used in refinement	92698 (9216)	57239 (5698)
Reflections used for R-free	4599 (427)	2886 (278)
R-work	0.1756 (0.2657)	0.2640 (0.3335)
R-free	0.2089 (0.2966)	0.2957 (0.3563)
CC(work)	0.948 (0.750)	0.874 (0.603)
CC(free)	0.940 (0.807)	0.844 (0.503)
Number of non-hydrogen atoms	9056	8782
macromolecules	7970	7925
ligands	156	136
solvent	930	721
Protein residues	971	970
RMS(bonds)	0.014	0.013
RMS(angles)	1.75	1.69
Ramachandran favored (%)	97.38	97.06

Ramachandran allowed (%)	2.41	2.94
Ramachandran outliers (%)	0.21	0.00
Rotamer outliers (%)	1.36	0.11
Clashscore	6.19	4.45
Average B-factor	28.92	33.83
macromolecules	27.61	33.21
ligands	38.17	53.55
solvent	38.59	36.90

```
Casp-1      SVGVSGNLSLPTTEEFEDDAIKKAHIEKDFIAFCSSTPDNVSWRHPTMGSVFIGRLIEHMQE
Casp-4      -------------------AVYKTHVEKDFIAFCSSTPHNVSWRDSTMGSIFITQLITCFQK
Casp-5      ---SLALISSQSSENLEADSVCKIHEEKDFIAFCSSTPHNVSWRDRTRGSIFITELITCFQK
Casp-2      -------------AGKEKLPKMRLPTRSDMICGYACLKGTAAMRNTKRGSWYIEALAQVFSE
Casp-8      ---------------LSSPQTRYIPDEADFLLGMATVNNCVSYRNPAEGTWYIQSLCQSLRE
Casp-10     ---------ALNPEQAPTSLQDSIPAEADFLLGLATVPGYVSFRHVEEGSWYIQSLCNHLKK
Casp-9      -------------------AISSLPTPSDIFVSYSTFPGFVSWRDPKSGSWYVETLDDIFEQ
Casp-6      -----------------AASVYTLPAGADFLMCYSVAEGYYSHRETVNGSWYIQDLCEMLGK
Casp-3      ------------SGVDDDMACHKIPVEADFLYAYSTAPGYYSWRNSKDGSWFIQSLCAMLKQ
Casp-7      -----------------------------FLFAYSTVPGYYSWRSPGRGSWFVQALCSILEE

Casp-1      YA-CSCDVEEIFRKVRFSFEQP--------------DGRAQMPTTERVTLTRCFYLFPGH--
Casp-4      YS-WCCHLEEVFRKVQQSFETP--------------RAKAQMPTIERLSMTRYFYLFPGN--
Casp-5      YS-CCCHLMEIFRKVQKSFEVP--------------QAKAQMPTIERATLTRDFYLFPGN--
Casp-2      RA-CDMHVADMLVKVNALIKDREG-----YAPGTEFHRCKEMSEYC-STLCRHLYLFPGHPP
Casp-8      RCPRGDDILTILTEVNYEVSNK---------DDKK-NMGKQMPQPT-FTLRKKLVFPSD---
Casp-10     LVPRMLKFLEKTMEIRGRKRTVWGAKQISATSLPT-AISAQTPRPP-MRRWSSVS-------
Casp-9      WA-HSEDLQSLLLRVANAVSVKG--------------IYKQMPGCF-NFLRKKLFFKTS---
Casp-6      YG-SSLEFTELLTLVNRKVSQRRVD----FCKDPSAIGKKQVPCFA-SMLTKKLHFFPKSN-
Casp-3      YA-DKLEFMHILTRVNRKVATEFES----FSFDATFHAKKQIPCIV-SMLTKELYFYH----
Casp-7      HG-KDLEIMQILTRVNDRVARHFES----QSDDPHFHEKKQIPCVV-SMLTKELYFS-----
```

Supplementary Figure 1: Conservation of C264. Multiple sequence alignment of the small subunit of the human caspases shows that C264 is not conserved between caspase-6 and the other caspases. Cysteine residues are shown in red and the green box indicates C264 in caspase-6. Sequence alignment conducted in clustal omega software provided by the European Molecular Biology Laboratory's European Bioinformatics Institute (EMBL-EBI).

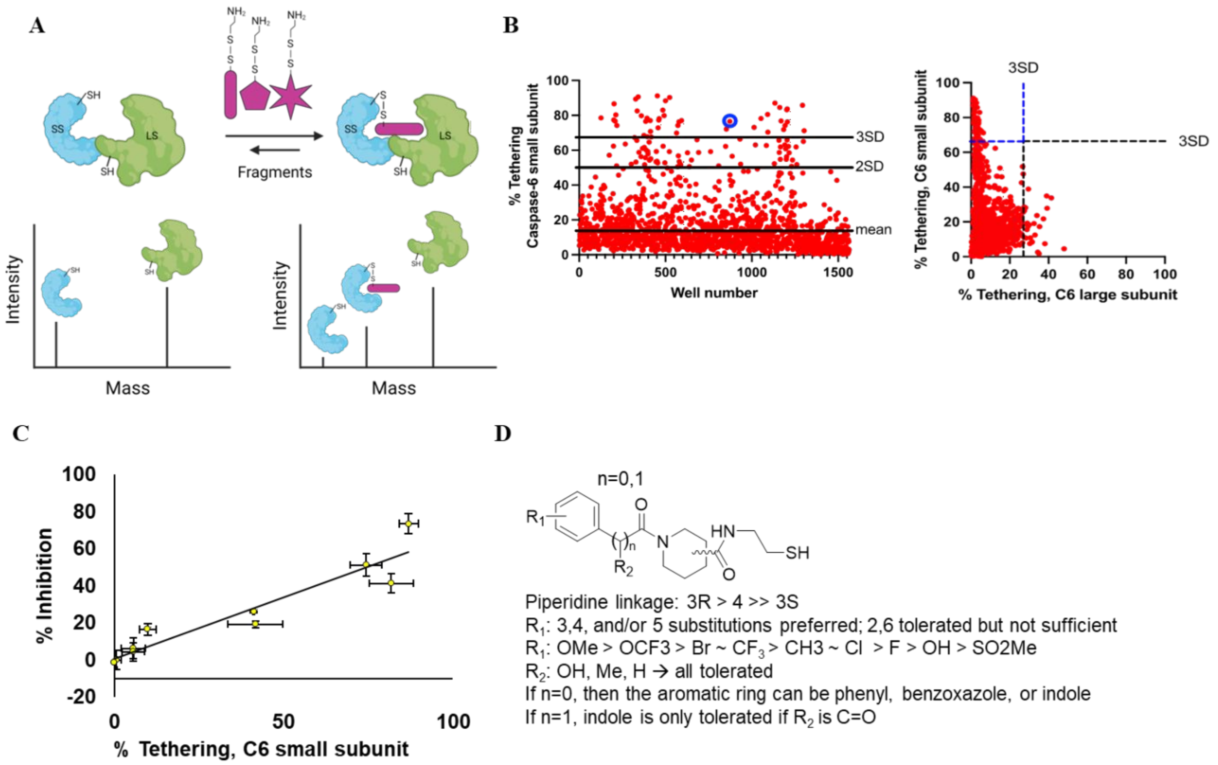

Supplementary Figure 2: Disulfide tethering screen. **A)** carton representation of disulfide tethering screen. Green represents the caspase-6 large subunit, blue the small subunit, and pink the disulfide fragments. **B)** results of the tethering screen with hits defined as being 3 standard deviations (SD) above the mean (top panel) with the lead fragment (**1**) circled in blue. Small subunit hits were compared to large subunit hits from the same screen (bottom panel). Generally, SS hits were stronger than LS hits. **C)** correlation of biochemical activity with % tethering for **1** showing a strong correlation (R= 0.9491). **D)** SAR of small subunit fragment hits.

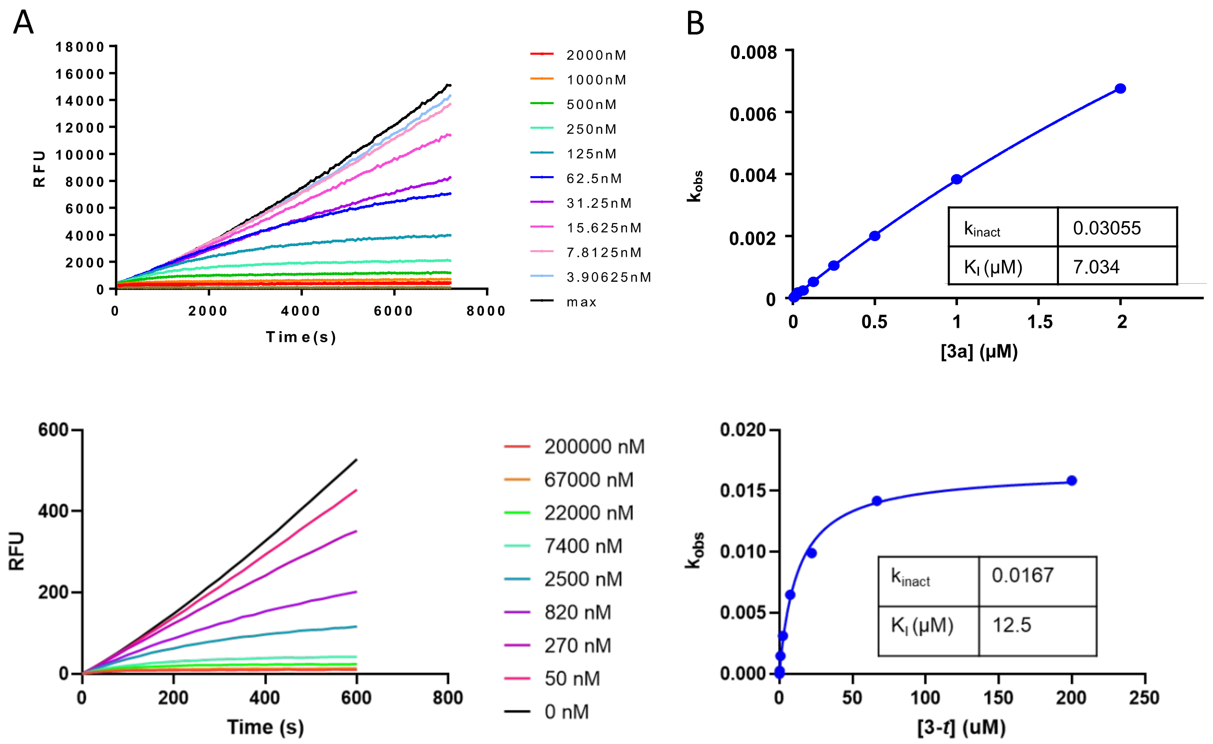

Supplementary Figure 3: measurement of kinact/Ki for 3a and (±)-3-t. A) time curves for C6 activity at the indicated concentrations of **3a** (top) and **3-t** (bottom). [C6] = 0.15 nM; [substrate] = 2 μM. **B)** Plot of kobs vs [**3a**] (top) and kobs vs [**3-t**] (bottom). kobs calculated from time curves from the formula: $[P] = \frac{v_i}{k_{\text{obs}}}(1 - e^{-k_{\text{obs}}\,t})$

c) kinact and Ki calculated from the formula:

$$k_{\text{obs}} = \frac{k_{\text{inact}} \times [I]}{K_{\text{I}} + [I]}$$

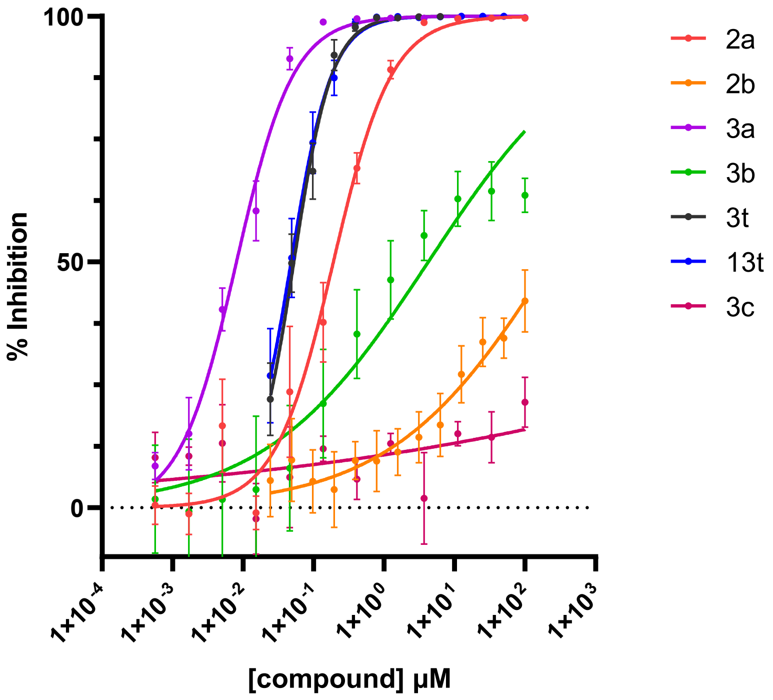

Supplementary Figure 4: Caspase-6 biochemical IC$_{50}$ data. Caspase-6 was subjected to various concentrations of the indicated inhibitors and a rhodamine based reporter peptide. The %inhibiton for each compound realitive to a vehicle control and known inhibitor (VEID-FMK) was plotted vs the concentration of the compound and an IC$_{50}$ was for each compound was determined in graphpad prism. Error bars represent the standard devation of each measurement with an n of 3.

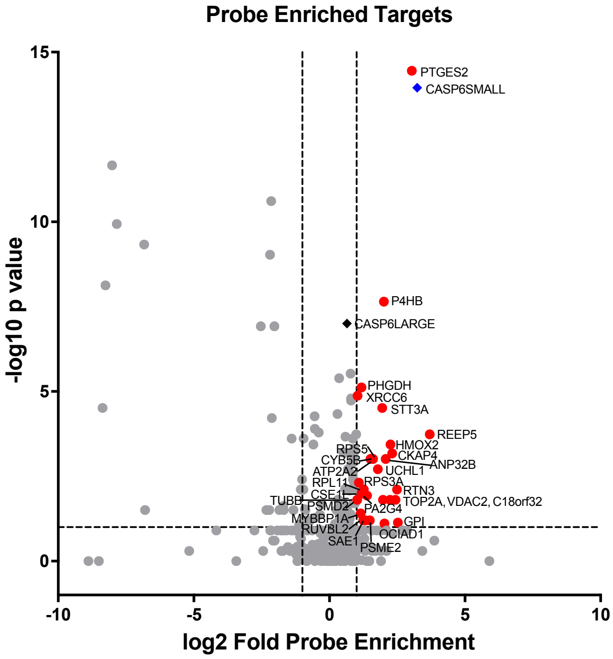

Supplementary Figure 5: SILAC quantitative proteomics. HEK293T cells transiently overexpressing caspase-6 were treated with 1 μM **13-*t***. Data represent enriched proteins compared to no-treatment controls in replicate samples and include 346 unique proteins, 88 of which had significant SILAC enrichment ratios (see methods). Red circles, enrichment > log2 and p value < 0.05; blue diamond, small subunit of caspase-6. Keratin proteins (KRT) are not highlighted because these proteins are likely present due to contamination.

Scan of ^{1}H NMR of compound 3a. (identical to **(±)-3-*t*** as expected)

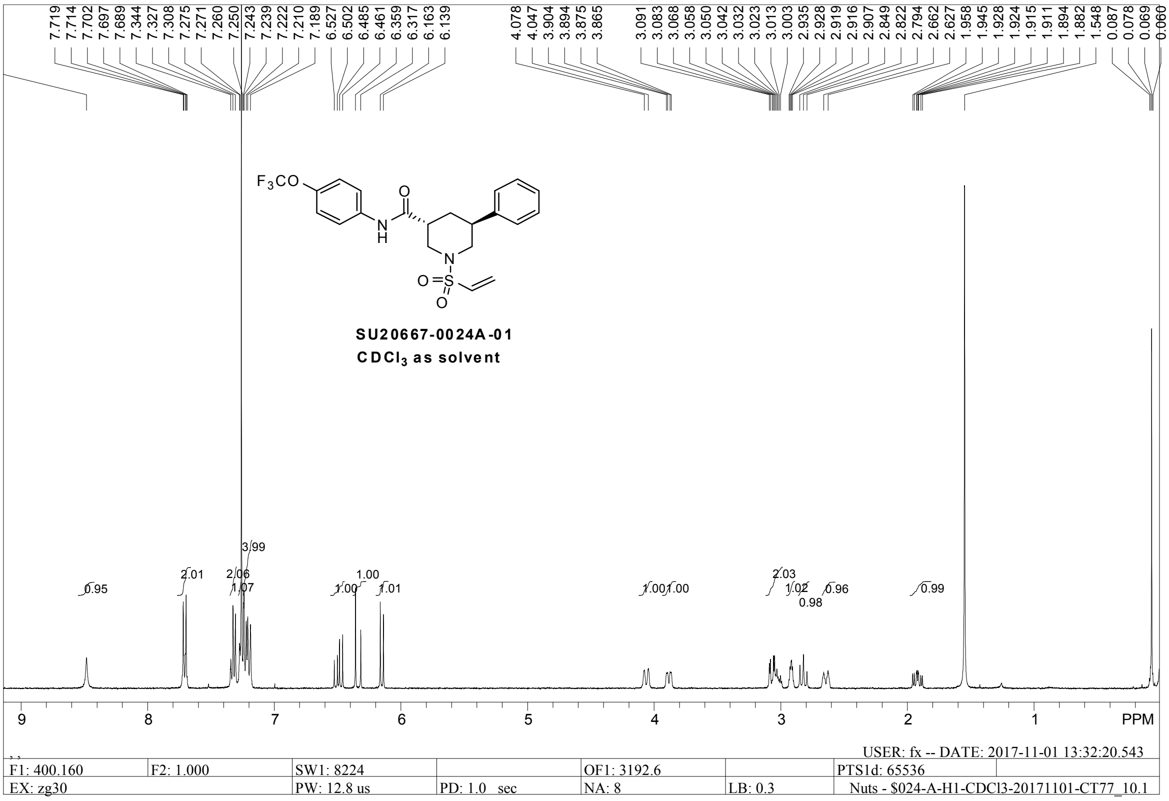

Scan of ^{1}H NMR of compound 3b. (identical to **(±)-3-*t*** as expected)

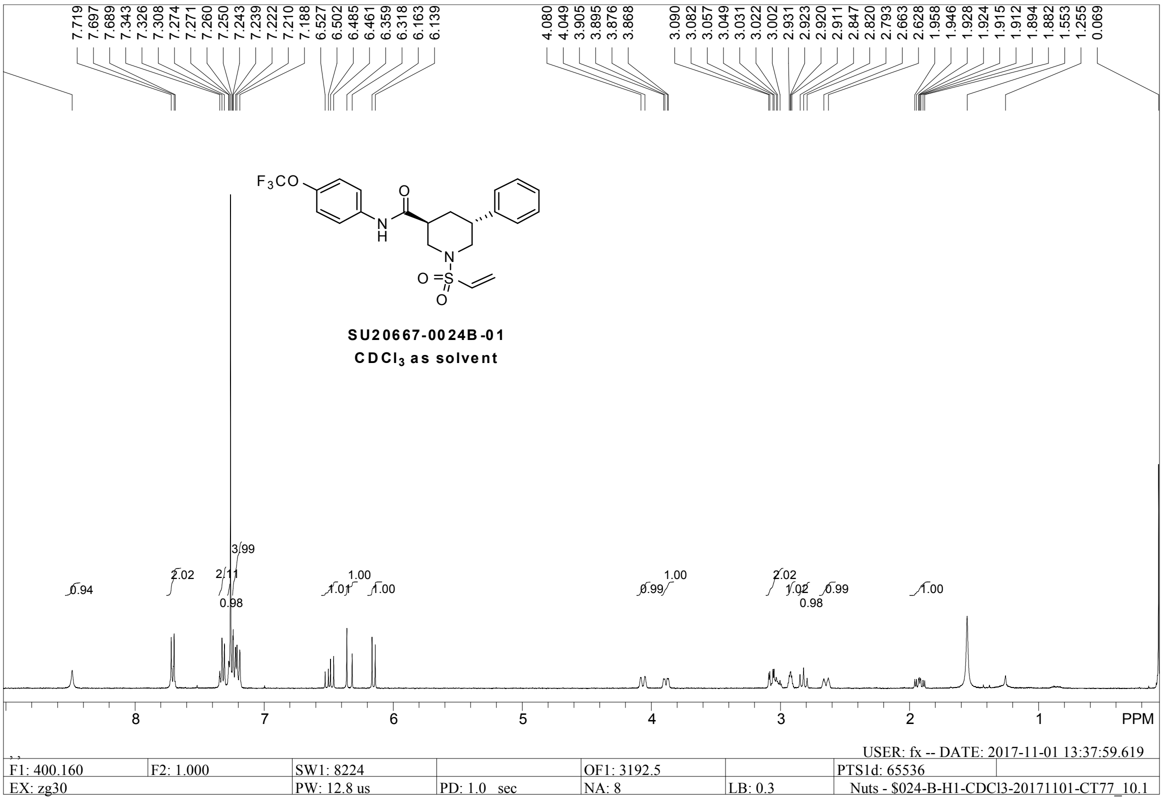

1H NMR Spectra of compound 3c. (identical to **(±)-3-*c*** as expected)

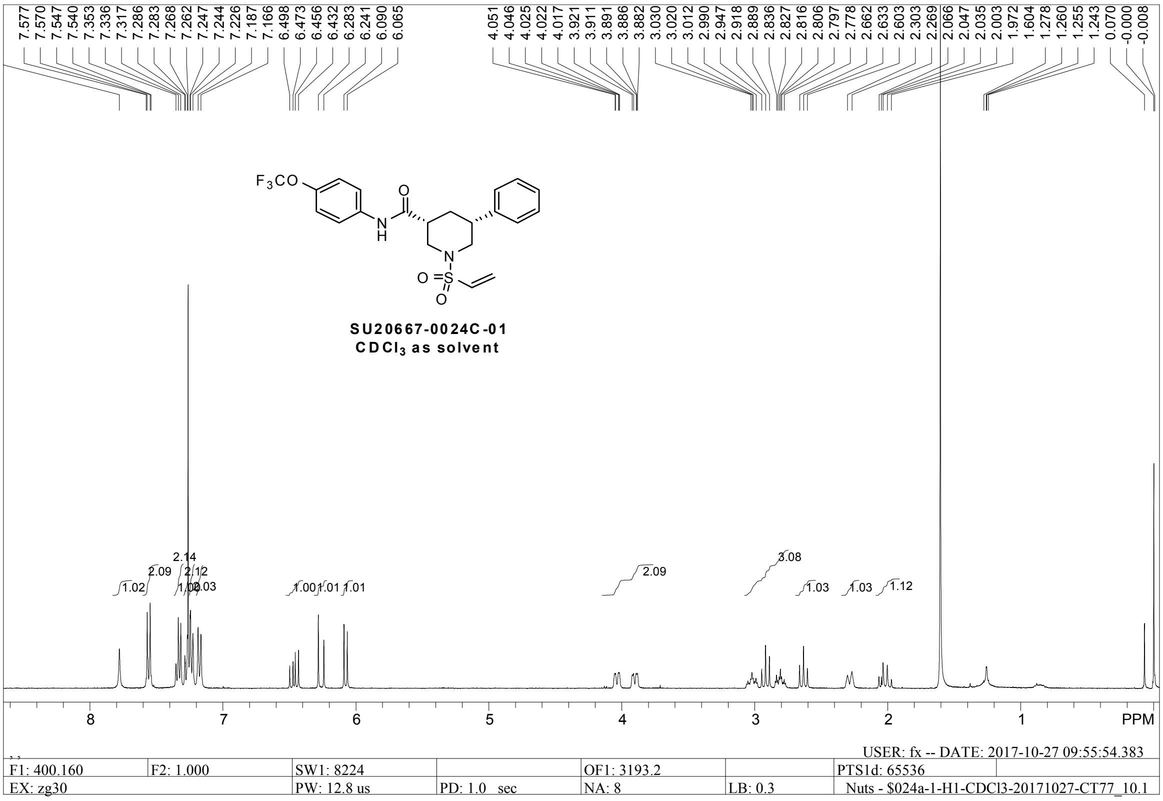

1H NMR Spectra of compound 3d. (identical to **(±)-3-*c*** as expected)

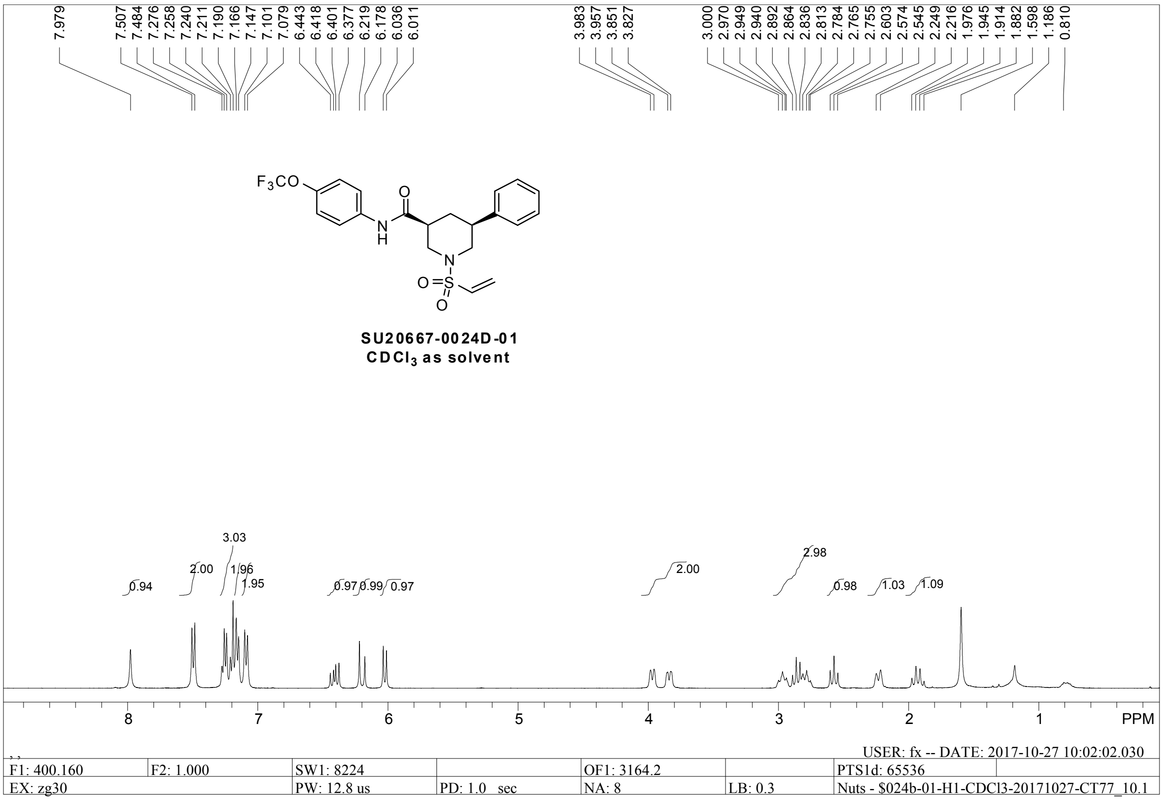

Supplementary Information References

(1) Burlingame, M. A.; Tom, C. T. M. B.; Renslo, A. R. *ACS Comb. Sci.* **2011**, *13*, 205.

(2) Turner, D. M.; Tom, C. T. M. B.; Renslo, A. R. *ACS Comb. Sci.* **2014**, *16*, 661.

(3) Heise, C. E.; Murray, J.; Augustyn, K. E.; Bravo, B.; Chugha, P.; Cohen, F.; Giannetti, A. M.; Gibbons, P.; Hannoush, R. N.; Hearn, B. R.; Jaishankar, P.; Ly, C. Q.; Shah, K.; Stanger, K.; Steffek, M.; Tang, Y.; Zhao, X.; Lewcock, J. W.; Renslo, A. R.; Flygare, J.; Arkin, M. R. *PLoS One* **2012**, *7*, e50864.

(4) Vagin, A. A.; Steiner, R. A.; Lebedev, A. A.; Potterton, L.; McNicholas, S.; Long, F.; Murshudov, G. N. *Acta Crystallogr. Sect. D, Biol. Crystallogr.* **2004**, *60*, 2184.

(5) Emsley, P.; Cowtan, K. *Acta Crystallogr. Sect. D, Biol. Crystallogr.* **2004**, *60*, 2126.

(6) Orth, K.; Chinnaiyan, A. M.; Garg, M.; Froelich, C. J.; Dixit, V. M. *J. Biol. Chem.* **1996**, *271*, 16443.

(7) Ruchaud, S.; Korfali, N.; Villa, P.; Kottke, T. J.; Dingwall, C.; Kaufmann, S. H.; Earnshaw, W. C. *EMBO J.* **2002**, *21*, 1967.

(8) Takahashi, A.; Alnemri, E. S.; Lazebnik, Y. A.; Fernandes-Alnemri, T.; Litwack, G.; Moir, R. D.; Goldman, R. D.; Poirier, G. G.; Kaufmann, S. H.; Earnshaw, W. C. *Proc. Natl. Acad. Sci. USA* **1996**, *93*, 8395.

(9) Mintzer, R.; Ramaswamy, S.; Shah, K.; Hannoush, R. N.; Pozniak, C. D.; Cohen, F.; Zhao, X.; Plise, E.; Lewcock, J. W.; Heise, C. E. *PLoS One* **2012**, *7*, e30376.

(10) Byrnes, J. R.; Weeks, A. M.; Shifrut, E.; Carnevale, J.; Kirkemo, L.; Ashworth, A.; Marson, A.; Wells, J. A. *Mol. Cell Proteomics* **2022**, *21*, 100217.

(11) Leung, K. K.; Wilson, G. M.; Kirkemo, L. L.; Riley, N. M.; Coon, J. J.; Wells, J. A. *Proc. Natl. Acad. Sci. USA* **2020**, *117*, 7764.

(12) Pino, L. K.; Searle, B. C.; Bollinger, J. G.; Nunn, B.; MacLean, B.; MacCoss, M. J. *Mass Spectrom Rev* **2020**, *39*, 229.
